# Isotopic reconstruction of short to absent breastfeeding in a 19th century rural Dutch community

**DOI:** 10.1371/journal.pone.0265821

**Published:** 2022-04-13

**Authors:** Andrea L. Waters-Rist, Kees de Groot, Menno L. P. Hoogland

**Affiliations:** 1 Department of Anthropology, University of Western Ontario, Ontario, Canada; 2 Faculty of Archaeology, Leiden University, North Holland, The Netherlands; 3 Historical Society of Beemster, North Holland, The Netherlands; 4 Koninklijk Instituut voor Taal, Land en Volkenkunde (KITLV), Royal Netherlands Institute of Southeast Asian and Caribbean Studies, Leiden, The Netherlands; University of Padova: Universita degli Studi di Padova, ITALY

## Abstract

Artificial feeding of young infants is considered a modern phenomenon. However, historical sources note its practice in some past European populations. This research explores factors that contributed to a short to absent period of breastfeeding in pre-modern Netherlands. Stable nitrogen and carbon isotope analysis is undertaken on 277 19^th^ century individuals from Beemster, a, rural, mainly Protestant, dairy farming community. An intra-individual sampling approach for ≤6 year-olds compares newer metaphyseal to older diaphyseal long bone collagen to classify feeding status at death. Archivally identified individuals permit analyses of sex and year-of-death. Few infants have isotopic evidence for breastfeeding and, if present, it was likely of short duration or a minor source of dietary protein. From only a few weeks to months of age infants were fed cow’s milk and paps with sugar. There is broad dietary homogeneity with no sex or temporal isotopic differences, but young infants (1–11 months) have the most ẟ^15^N and ẟ^13^C variation highlighting the effect of multiple dietary and physiological processes. Beemster had many factors associated with high rates of breastfeeding in other Dutch communities, yet, most mothers did not breastfeed, or not for long, showing that regional variation in infant feeding is influenced by community values and traditions. On top of child rearing and domestic chores, female dairy farmers were in charge of milking cattle and dairy production, an important income source, suggesting high workload was also a factor in short or absent breastfeeding periods, aided by the constant supply of fresh milk that could be fed to an infant by an older sibling.

## Introduction

Bioarchaeology lacks studies of pre-modern populations with short to absent periods of breastfeeding largely because, until the advent of commercialized formulas, most infants were breastfed at least until they were ready for other foods beginning around 4 to 6 months-of-age. Thereafter, mixed maternal and non-maternal feeding often continued for several years during the process of weaning. The archaeological record contains infants who were not breastfed, perhaps because of maternal death [e.g., 1], but research thus far has found this was the exception, not the rule. After all, breastmilk is the perfect food for infants (at least until ~6 months of age) because it is nutritious, sterile, confers immunity, promotes gastrointestinal maturation and health, and during the period of exclusive breastfeeding, prevents the infant from ingesting contaminated foods.

Isotopic reconstructions of infant feeding practices from a wide geographic and temporal range of past populations have found weaning ceased between one- to six-years of age, with an average around two to three-years [2, 3]. The shortest breastfeeding periods have been found in European post-Medieval populations (from roughly CE 1500–1900) where weaning was complete around the first year [4, 5]. These populations came from large urban centres and one included a lower-class rapidly industrializing area where mothers worked outside the home; these factors are often noted to have caused a decline in breastfeeding in historical times [6–10]. Technological advancements in infant feeding devices made during the post-Medieval period, and the widening availability of animal milks, enabled artificial feeding [9–12]. Devices included buddy-pots and pap boats. The former were like a small coffee-pot with the neck coming from the bottom and a spout with small holes over which a cloth was tied through which the infant could suck milk, and the latter held variable mixtures of ‘water-milk-broth-bread-flour-grain’ and included a spoon with a hollow stem through which food could be blown down the infant’s throat [10, 12–14]. Another simple, common feeding method was to spoon-feed the infant animal milk or even have them drink directly from the teat. Cow’s horns and clay and wood vessels have also been used as infant feeding devices. Glass bottles were invented in 1851 and advances in rubber production made odorless rubber nipples available by the latter part of the 19^th^ century [10–13].

Infant feeding was an understudied topic in historic times, but some texts discussed breastfeeding in the context of concerns about high infant mortality rates (IMR). In 19^th^ century Netherlands, the focus of this study, around one in four or five infants died in their first year [15–20]. This prompted investigations into the causes of high IMRs, which found strong correlations with a lack of breastfeeding or its early cessation, combined with unsuitable breastmilk alternatives and/or other harmful weaning practices [15, 21–27]. This prompted several historical Dutch texts to strongly advocate for maternal breastfeeding [22, 25, 28–30].

For such a small country, the Netherlands had marked regional variation in IMR during the post-Medieval period, implying considerable variation in infant feeding practices [9, 16, 19, 20, 31, 32]. Several variables likely affected 19^th^ century Dutch breastfeeding practices. Debate surrounds the idea that Dutch Catholic communities had lower rates and shorter durations of breastfeeding (which resulted in higher IMRs) compared to Liberal Protestant communities (Liberal Protestant denominations consist of the Dutch Reformed (majority) and Mennonites, Lutherans, and Remonstrants (minority) and excludes Orthodox Protestant denominations such as Dutch Calvinism, which often had high infant mortality [33]) [9, 27, 31, 33–44]. Catholic ideology considered female nudity shameful such that breastfeeding should only take place in private and, in some communities, there was resurgence of a custom of binding young women’s breasts combined with dresses with tight bodices that could cause problems with lactation [39, 41, 42].

Occupations where mothers worked outside of the home were obviously the least conducive to breastfeeding, and this was most common in urban factory workers [9, 12, 32]. However, during harvest season, crop farmers wives often needed to work in fields far away from home, thus also limiting breastfeeding for at least part of the year [16, 45, 46]. Some studies found breastfeeding was common amongst poor or landless urban families because they could not afford or access other types of food like animal milk [47, 48]. Farmers and farm-workers often had amongst the lowest IMRs of different occupation groups [43, 49, 50] which has been used to infer a moderate to long breastfeeding period [32]. Farmers owned or leased the land upon which they lived and worked and are of higher socioeconomic status than landless farm-workers which includes permanent year-round employees and temporary short-term workers, the former having more stable positions with slightly higher wages with benefits. Yet, IMRs are influenced by a host of factors including maternal nutrition, maternal support (unmarried mothers had the highest IMRs of any group), family size and wealth, weaning food quality and age-of-introduction, and infectious diseases (related to water quality, sanitation, and the density of people in a living area), so are not necessarily a reliable method of inferring the length of breastfeeding [9, 25, 32, 44]. Infants of upper class mothers may have been fed by a wet-nurse, but the practice was never as popular in the Netherlands as in other western European nations [51, 52].

The role of dietary differences in Dutch infant feeding practices has not been explored. Common weaning foods included animal, mostly cow’s but also goat’s milk, often diluted with water, and pap, also called porridge, gruel, and panada, which was a liquid to semisolid food made from flour, bread or cereals and cooked in water, milk or broth; sugar was purportedly often added to these foods [13, 53] (pap, panada and gruel/porridge are actually different dishes, varying in consistency and the use of grains vs. flour/bread and broth vs. water/milk [13] but many authors use the terms synonymously). For the rest of the population the national staples were rye, wheat or barley bread, potatoes, oats, and vegetables like cabbage, carrots, peas, broad beans, endive, cucumber, and onion; diets usually contained little meat [20, 54]. Chicken eggs were a common source of animal protein [55]. Yet, there were regional dietary differences with the province of North Holland being a part of ‘Western Dutch cuisine’, characterized by its high production and consumption of bovine dairy products, especially cheese, butter, and buttermilk [54, 56]. The commonality of dairy foods may have implications for infant feeding practices. Coastal provinces like North Holland also had access to more seafood [54].

**Fig 1:**
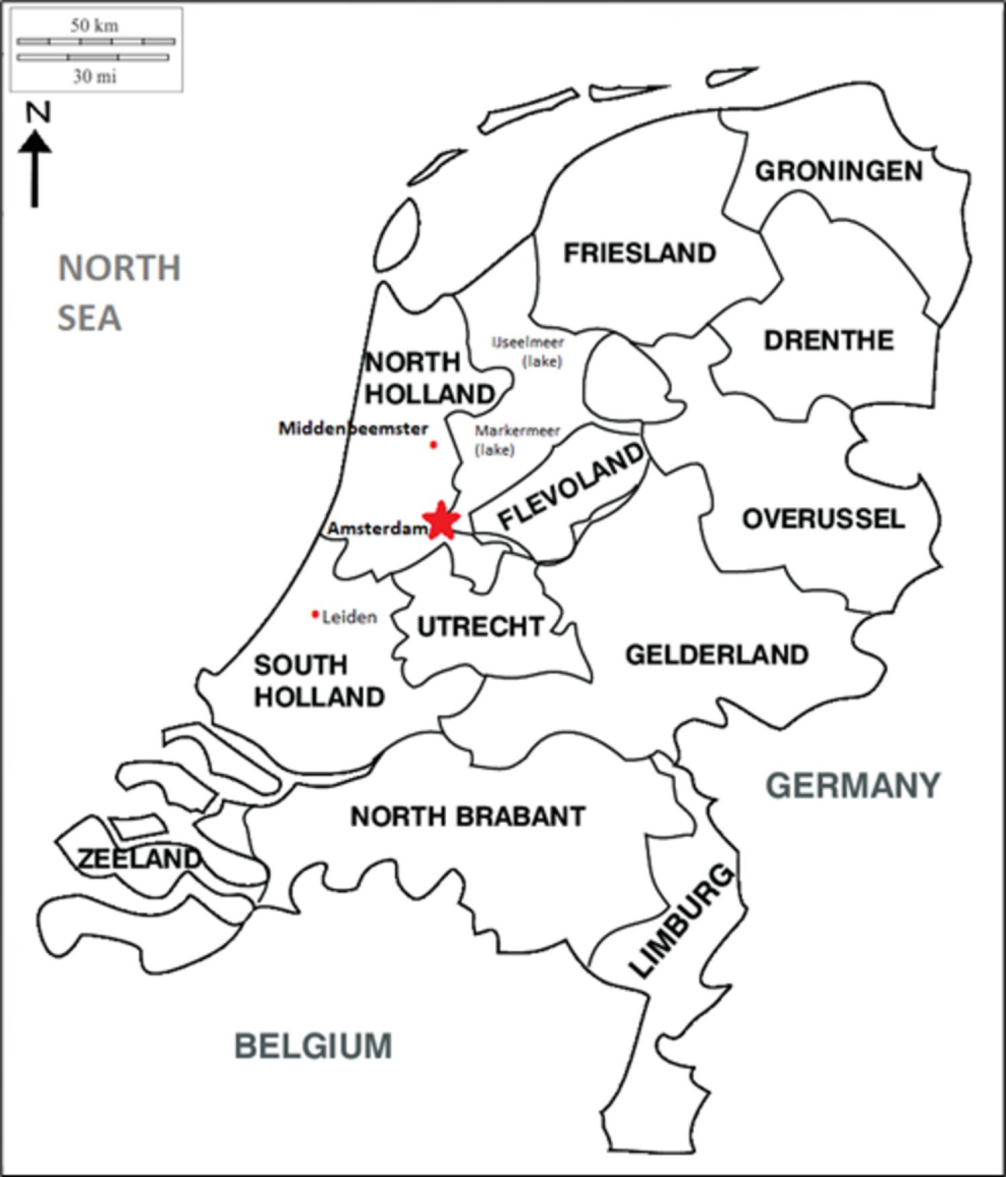
The Netherlands provinces showing location of the Middenbeemster village.

The objective of this study is to understand the breastfeeding and weaning practices of 19^th^ century rural villagers from Beemster, North Holland (Fig 1). Stable isotope analyses of bone collagen are used to elucidate the presence and approximate duration of breastfeeding and the common types of weaning foods. Stable nitrogen (ẟ^15^N) and carbon (ẟ^13^C) isotope ratios undergo, respectively, roughly three and one permil trophic level increases in a breastfeeding infant relative to its mother (or wet-nurse); a change that is absent in non-breastfed infants [57–62]. Many researchers have used this phenomenon to reconstruct the infant feeding patterns of past populations [63, 64]. Archival information permitting identification of a subset of Beemster individuals is used to explore if there were differences in infant feeding according to sex and time-period from 1830 to 1867. Infant feeding patterns are then discussed to elucidate the key factors that affected breastfeeding and weaning behaviors in Beemster.

## Materials

This research concerns 19^th^ century inhabitants of the rural Beemster polder (land reclaimed ‘from the sea’) (Fig 1). The post-Medieval economy of North Holland was based on international trade, financial services, and in the rural regions like Beemster, cattle breeding and dairy farming [65]. Dairy farms had on average 20 cows and focussed on quality goods for export, such as butter and cheese [66]. The sale and price of these exports, as well as cattle, increased substantially in the 1800s, making dairy farming a generally reliable and profitable occupation [66, 67]. Farms were run by families as knowledge about the animals, pastures, and market products was passed down through the generations and there was room for several children to hold important roles in the business [68]. Wives were in charge of dairy production and, with children of suitable age, would milk cows, churn butter, and make buttermilk and cheese [69]. Traditional forms of labor and methods of food preparation would have been followed as the site preceded industrialization of the farming industry in the Netherlands [70].

Middenbeemster is the principle village of the Beemster. It contains the Dutch Reformed Protestant Keyser Church, completed in 1623. The village of Westbeemster was a small Catholic enclave within the polder. It lacked its own Church and graveyard until 1848; before that both Catholics and Protestants were buried at the Protestant Church. At the request of the community, in 2011 approximately 500 individuals were excavated from the Protestant Church cemetery by Hollandia Archeologen and Leiden University, Faculty of Archaeology. Civil and church registers show most individuals were buried between 1830 to 1867 (when cemetery use ceased). The practice of wealthier individuals paying to be buried inside a Church was prohibited in 1829, so the analyzed sample contains all socioeconomic groups [69, 71]. For the archival identification of individuals, parish registration records are linked to the municipal registration of death (Begraafregister) and civil registration records of vital events.

Stable isotope analysis is performed on 277 individuals, of which 185 (67%) are identified by name, age-at-death, and year-of-death. Table 1 shows this information per age category. Standard osteological methods were used to estimate the age-at-death and sex (male = M; female = F) of individuals who could not be archivally identified [detailed in 72]. Sex was only estimated for adults (≥18 years) and adults of indeterminate sex are not included in this research.

**Table 1:** Number of analyzed individuals from Beemster per age category.

Age Range	Number of Individuals	Number with Archival ID
**Fetal & Neonate** (*in utero–* 28 days)	27	15
**Young Infant** (1–11 months)	23	9
**Older Infant** (1 year)	5	3
**Toddler** (2 years)	9	6
**Child** (3–6 years)	23	15
**Juvenile** (7–12 years)	25	12
**Adolescent** (13–17 years)	13	5
**Young Adult (YA) F** (18–34 years)	31	26
**Young Adult (YA) M** (18–34 years)	17	13
**Middle Adult (MA) F** (35–49 years)	16	11
**Middle Adult (MA) M** (35–49 years)	18	8
**Old Adult (OA) F** (50+ years)	33	31
**Old Adult (OA) M** (50+ years)	37	31
**TOTAL**	**277**	**185**

## Methods

Ethical approval was granted by the Social Sciences and Humanities Ethics Committee of Leiden University. No permits were required for the described study, which complied with all relevant regulations. A full list of specimen numbers are provided in S2 Table. Skeletons are stored at the Laboratory for Human Osteoarchaeology, Faculty of Archaeology, Leiden University, The Netherlands.

Human ẟ^15^N and ẟ^13^C values reflect the isotopic composition of their diet and can be used to estimate the proportion of terrestrial vs. marine foods, plants that use different photosynthetic pathways (C3 vs. C4), and the trophic level of consumed protein [62, 73, 74]. ẟ^15^N values can also be affected by malnutrition or illness that causes tissue catabolism and it is critical to consider this, rather than breastfeeding, as a cause of higher ẟ^15^N values in infants and children (and to distinguish the two causes of ^15^N enrichment if possible) [75–79]. The plants in the Dutch diet follow a C3 photosynthetic pathway, associated with lower ẟ^13^C values, with one possible exception: the C4 plant sugarcane (as opposed to sugar beet which is a C3 plant) [20, 80].

Dietary interpretations should be done using the isotopic values of the animals and plants that were likely consumed by humans. Faunal and floral remains were not found at the Middenbeemster cemetery but published isotope data for terrestrial and marine animals from five Dutch Medieval sites are used to aid in the interpretation of human results [81–84] (S1 Table). Bone collagen ẟ^15^N values only reflect the protein component of the diet; δ^13^C values are also largely reflective of protein (~three-quarters) with smaller contributions from carbohydrates and lipids (~one-quarter) [85, 86].

Intra(within)-individual sampling, from different mineralized tissues or between different parts of the same anatomical element, is useful for reconstructing an individual’s diachronic dietary history. This study compares ẟ^15^N and ẟ^13^C values from the metaphyseal area of a growing long bone with those from the diaphysis of the same bone in all individuals ≤6-years-of-age (n = 87). The long bones studied herein begin formation in the seventh to eighth week of fetal life, with the bone shaft, the diaphysis, mineralizing first [87]. Growth in length (longitudinal growth) then occurs at the ends of the long bone, under the growth plate located between the end of the bone shaft, the metaphysis, and the unfused secondary growth centre, the epiphysis. Thus, the metaphyses contain tissue formed most recently before death, while the diaphysis contains tissue formed at an earlier age [88, 89]. Rates of bone growth and remodelling will determine how much bone tissue in a particular area was formed during any particular time-period before death [90]. All metaphyseal samples were taken from the end of the bone that undergoes the most growth (proximal humerus, distal radius and ulna, distal femur, and proximal tibia [91, 92]) while diaphyseal samples were collected from the area of the primary nutrient foramen (S2 Table).

Bone samples for juveniles (7–12 years), adolescents (13–17 years) and adults (18+ years) were taken from a rib body when available; if not available, a long bone fragment containing some trabecular bone or a small phalanx were used (S2 Table). These bone areas should reflect an individual’s diet over the last 5 to 10 or more years of life with trabecular bone typically containing more younger tissue than cortical bone [90, 93, 94]. A total of 715 samples were analyzed. Statistical analyses are performed in SPSS v25.

Samples were manually cleaned and then washed ultrasonically in distilled H_2_O. Collagen was extracted following the ‘Sealy method’ [95] by immersion in dilute HCl (0.5%), which was changed every 24–48 hours until demineralization was complete. A 0.125% solution of NaOH was applied for 20 hours to remove humic contaminants. Finally, the sample was rinsed and soaked in distilled H_2_O until reaching a neutral pH and then freeze-dried.

Collagen samples were combusted using a Thermo-Scientific Flash 2000 organic elemental analyser with the resultant gases introduced into the Delta V plus isotope ratio mass spectrometer via a continuous flow (Conflo) inlet located in the Stable Isotope laboratory, Vrije Universiteit, Amsterdam. Isotopic values are reported as ẟ values in permil (‰). ẟ^13^C values are reported relative to the Vienna PeeDee Belemnite (VPDB) marine limestone standard, and ẟ^15^N values are reported relative to the international nitrogen standard, air. Internal reference standards (USGS40, USG41, USG42, glycine) and ~5% of samples analyzed in duplicate or triplicate were used to determine that the precision of the mass spectrometer was 0.2‰ for ẟ^13^C and ẟ^15^N. All samples had acceptable collagen yields that varied from 1.7 to 27.0%, atomic C/N ratios between 3.0 to 3.5, and %N and %C by weight values between 12.3–18.2 and 35.6–52.2, respectively (S2 Table), suggesting the lack of post-mortem alteration [96–98].

**Fig 2:**
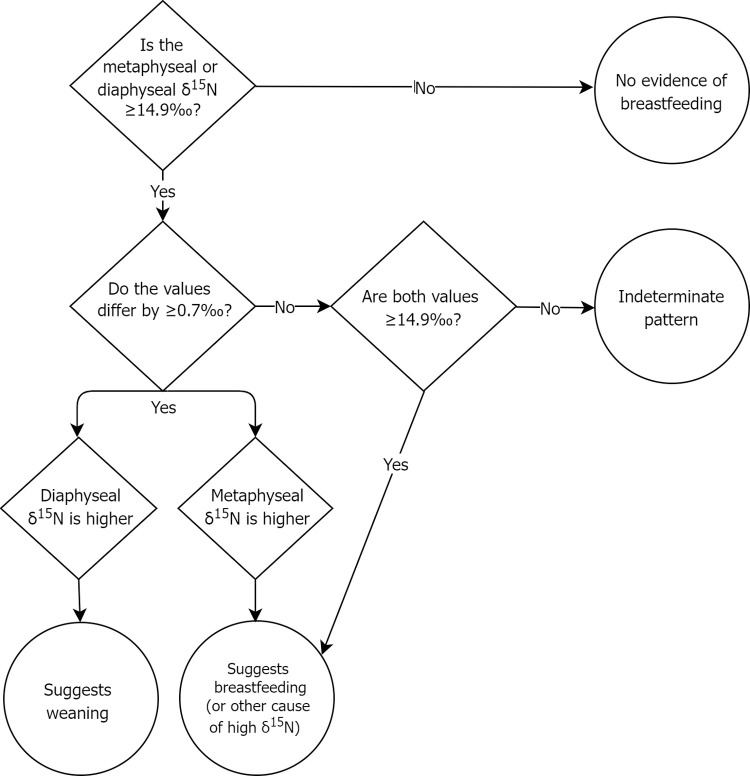
Interpretive method 1 decision tree for categorizing infant feeding status at death from paired metaphyseal and diaphyseal ẟ^15^N values.

Two methods are used interpret the patterning of isotope values of the ≤6-year-olds to suggest their feeding status at death, where the pattern is either consistent with breastmilk consumption with or without evidence of weaning (i.e. classified as weaning or breastfeeding, respectively) or the lack of detectable breastmilk consumption (i.e. classified as no breastfeeding). Fig 2 shows how the metaphyseal-diaphyseal ẟ^15^N values for ≤6-year-olds are interpreted using interpretive method 1. To establish that an infant’s ẟ^15^N values are possibly reflective of breastfeeding, one must take into account the ẟ^15^N data of both the fetuses/neonates (n = 52; $\bar{x}$ = 14.1‰; SD = 0.7‰) and adult Fs of reproductive age (includes young adults (YA) and middle adults (MA); n = 47; $\bar{x}$ = 13.7‰; SD = 0.7‰). To do so, we use the standard deviations (SD) of these groups as a conservative marker of meaningful dietary difference. As the fetal/neonate mean is higher, its mean (14.1‰) plus one SD (0.7‰), equalling 14.8‰, establishes that ẟ^15^N values ≥14.9‰ are possibly suggestive of the nursing trophic effect. Using the same SD of 0.7‰, one can infer if the individual’s predominate protein source had changed from breastmilk to weaning foods (with lower ẟ^15^N values). If the newer bone in the metaphysis has a ẟ^15^N value that is at least 0.7‰ higher than the older bone in the diaphysis (with the lower diaphyseal value reflecting bone formed *in utero*), this suggests breastmilk may have been the predominate protein source and the individual is classified as ‘breastfeeding’. In contrast, if the metaphyseal ẟ^15^N value is lower by 0.7‰ or more than the diaphyseal value, this is suggestive of weaning (with the higher diaphyseal value having accrued during breastfeeding). If the metaphyseal and diaphyseal ẟ^15^N values do not differ by 0.7‰ but are both ≥14.9‰, this suggests the entire bone is reflecting breastfeeding or another cause of high ẟ^15^N. Finally, if only one of the sampling locations has a ẟ^15^N value ≥14.9‰, but the two values do not differ by 0.7‰, it is not possible to suggest feeding status, resulting in a classification of indeterminate.

It is important to keep in mind that the use of the fetal/neonate ẟ^15^N mean and SD could be imperfect because these individuals are non-survivors and some of their isotope values may reflect poor maternal-infant health and physiological stress experienced *in utero*. Acute causes of death, such as birth obstruction/complications, which were fairly common in 19^th^ century Netherlands [99], would not lead to altered bone collagen ẟ^15^N values but it is not possible to distinguish acute from chronic conditions and the two may have co-occurred. However, we retain use of the threshold of ‘fetal/neonate ẟ^15^N mean + 1 SD’ to identify breastmilk consumption because it is a better gauge of early life diet than the adult F mean and it avoids overinterpreting small isotopic differences. We acknowledge that breastfeeding (and hence weaning) may be underrepresented in our interpretations if there were breastfeeding individuals whose ẟ^15^N values did not exceed this conservative threshold.

**Fig 3:**
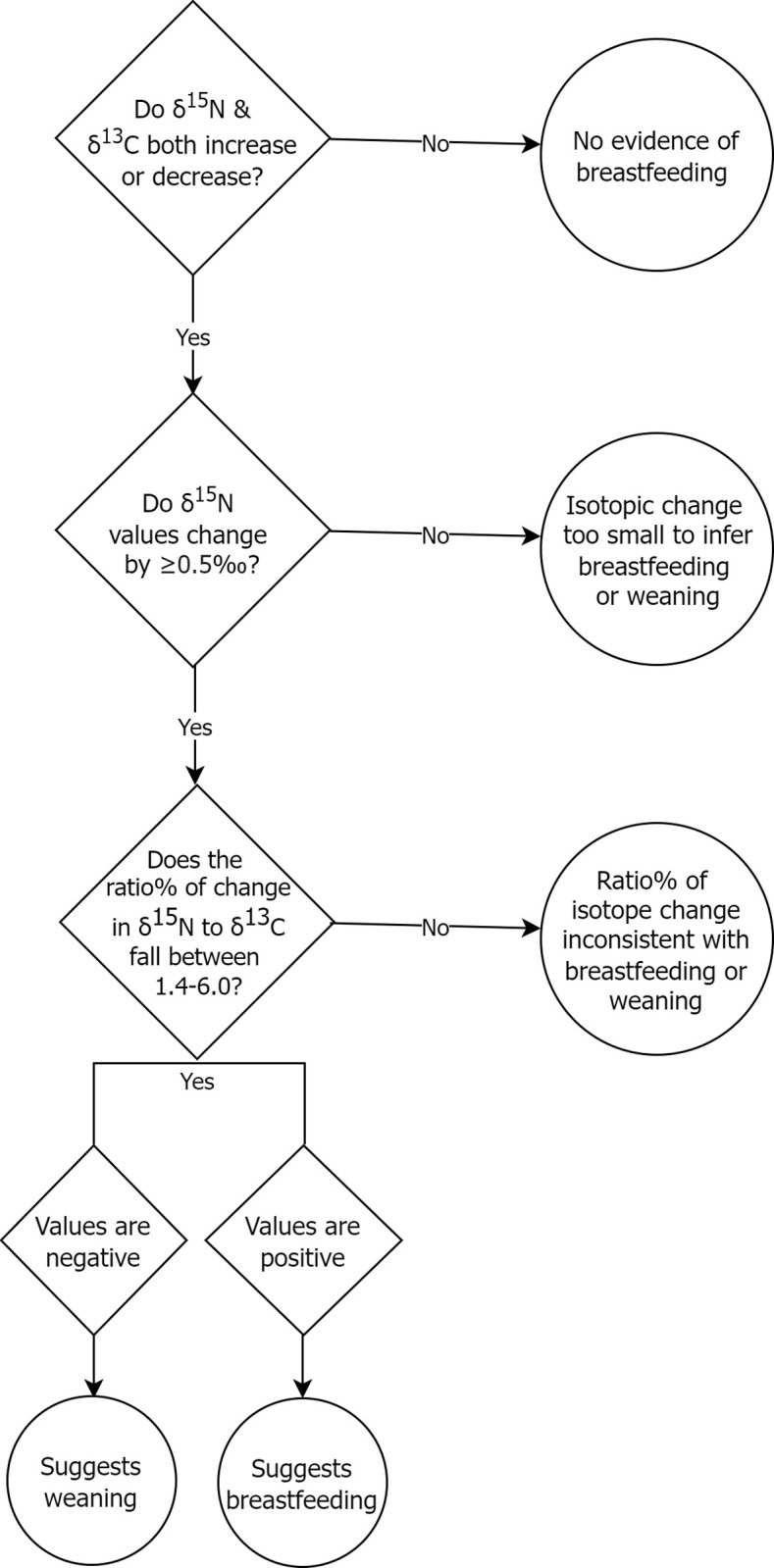
Interpretive method 2 decision tree for categorizing infant feeding status at death from covariation of ẟ^15^N and ẟ^13^C values.

Using the ẟ^15^N mean of the adult Fs or fetuses/neonates can be problematic because infants who were born from or breastfed by mothers with low ẟ^15^N values would in turn have low δ^15^N values compared to other infants and thus be categorized as ‘no breastfeeding’. To avoid this potential source of inaccuracy, interpretive method 2 instead considers the pattern of covariation of ẟ^15^N and ẟ^13^C values which has been shown to be a reliable indicator of breastfeeding and weaning when working with incremental dentine collagen samples [100, 101]. Method 2 thus examines the correspondence, extent, and ratio of isotopic change. As shown in Fig 3, first, it is noted if an individual’s diaphyseal to metaphyseal ẟ^15^N and ẟ^13^C values both increase (consistent with breastfeeding) or decrease (consistent with weaning). If not, the pattern is likely inconsistent with breastmilk consumption (whether exclusive or during weaning) and the classification is ‘no breastfeeding’. If yes, the pattern may be consistent with breastmilk consumption. To avoid overinterpreting minor isotopic variation arising from non-dietary factors (e.g., measurement imprecision, sample heterogeneity) the extent of the ẟ^15^N change must exceed the standard deviation of the maximum diaphyseal-metaphyseal difference of the fetal/neonate group, which is 0.4‰. Finally, if this criterion is met, the ratio of ẟ^15^N to ẟ^13^C change needs to be similar to the respective standard trophic shift of +3‰ to +1‰, thus 3:1 (or 3.0). As a certain amount of variation in this ratio is expected, especially during weaning when non-maternal foods will interfere with a pure breastfeeding signal, a ratio between 7:5 to 6:1 (or 1.4 to 6.0) is considered as possibly arising from breastfeeding or weaning while values outside this range are more likely due to other dietary changes or non-dietary factors.

## Results

**Fig 4:**
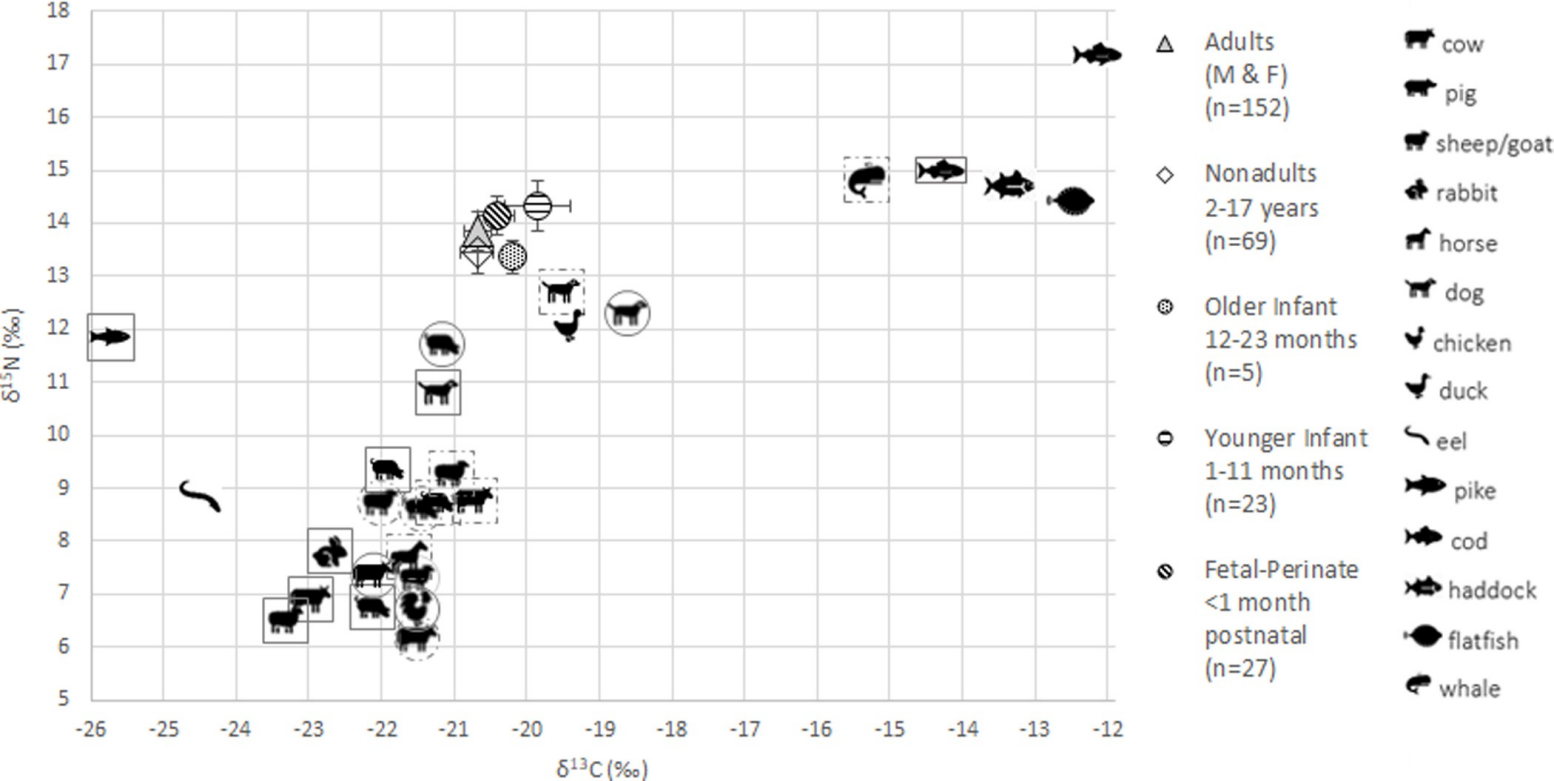
Scatterplot of certain Middenbeemster age-group ẟ^13^C and ẟ^15^N results with published archaeological faunal isotope results from five medieval Dutch sites. Symbols in solid square = site of Sint-Oedenrode [81]; dashed square = site of Oosterbeintum [83]; solid circle = site of Canadaplein, Alkmaar [82]; dashed circle = site of Doelenstraat, Alkmaar [82]; no border = site of Oldenzaal [84].

Note, S3 Table contains the results of statistical comparisons not mentioned here. Table 2 shows the mean ẟ^15^N and ẟ^13^C results for each age group and for the different sampling locations of the ≤6 year-olds. Adult M and F ẟ^15^N and ẟ^13^C values do not differ significantly (ẟ^15^N t = 0.352, p = 0.725; ẟ^13^C t = 0.516, p = 0.607). Fig 4 depicts these results (with certain age groups combined) with the published ẟ^15^N and ẟ^13^C values of archaeological fauna from five Dutch Medieval sites dating from CE 400–1573 (S1 Table).

**Table 2:** δ^15^N and δ^13^C results for the Middenbeemster cemetery.

Age Category	δ^15^N (‰)		δ^13^C (‰)	
	metaphysis	diaphysis	metaphysis	diaphysis
Fetal–Neonate (*in utero*—<1 month)	n: 25 $\bar{x}$: **14.0** SD: 0.78	n: 27 $\bar{x}$: **14.1** SD: 0.73	n: 25 $\bar{x}$: **-20.2** SD: 0.73	n: 27 $\bar{x}$: **-20.4** SD: 0.53
Young Infant (1–11 months)	n: 20 $\bar{x}$: **14.2** SD: 1.03	n: 23 $\bar{x}$: **14.3** SD: 0.94	n: 20 $\bar{x}$: **-19.8** SD: 1.18	n: 23 $\bar{x}$: **-19.8** SD: 0.89
Older Infant (1 year)	n: 4 $\bar{x}$: **13.2** SD: 0.37	n: 5 $\bar{x}$: **13.5** SD: 0.46	n: 4 $\bar{x}$: **-20.1** SD: 1.21	n: 5 $\bar{x}$: **-20.1** SD: 1.29
Toddler (2 years)	n: 9 $\bar{x}$: **13.6** SD: 0.66	n: 9 $\bar{x}$: **13.8** SD: 0.84	n: 9 $\bar{x}$: **-20.6** SD: 0.68	n: 9 $\bar{x}$: **-20.6** SD: 0.66
Child (3–6 years)	n: 23 $\bar{x}$: **13.4** SD: 0.83	n: 22 $\bar{x}$: **13.7** SD: 0.92	n: 23 $\bar{x}$: **-20.5** SD: 0.80	n: 22 $\bar{x}$: **-20.6** SD: 0.46
	mixed samples		mixed samples	
Juvenile (7–12 years)	n: 25 $\bar{x}$: **13.4** SD: 0.60		n: 25 $\bar{x}$: **-20.7** SD: 0.32	
Adolescent (13–17 years)	n: 13 $\bar{x}$: **12.9** SD: 0.88		n: 13 $\bar{x}$: **-20.8** SD: 0.45	
YA F (18–34 years)	n: 31 $\bar{x}$: **13.7** SD: 0.69		n: 31 $\bar{x}$: **-20.7** SD: 0.32	
YA M (18–34 years)	n: 17 $\bar{x}$: **13.6** SD: 0.78		n: 17 $\bar{x}$: **-20.8** SD: 0.37	
MA F (35–49 years)	n: 16 $\bar{x}$: **13.8** SD: 0.56		n: 16 $\bar{x}$: **-20.7** SD: 0.25	
MA M (35–49 years)	n: 18 $\bar{x}$: **13.7** SD: 0.61		n: 18 $\bar{x}$: **-20.6** SD: 0.35	
OA F (50+ years)	n: 33 $\bar{x}$: **14.1** SD: 0.55		n: 33 $\bar{x}$: **-20.6** SD: 0.48	
OA M (50+ years)	n: 37 $\bar{x}$: **14.0** SD: 0.97		n: 37 $\bar{x}$: **-20.7** SD: 0.40	

n = number; $\bar{x}$ = mean; SD = standard deviation.

Table 3 displays the results of the application of interpretive methods 1 and 2 for isotopic categorization of feeding status at death as shown in Figs 2 and 3. Both methods classify the majority of nonadults as ‘no breastfeeding’, and hence no weaning, however, otherwise the methods yield somewhat different results (denoted by italicized text in the last columns of table 3). We cannot determine which method is more accurate until ongoing incremental dentine isotope research is completed, however, we can use the fetal/neonate group as a gauge of a methods appropriateness given that all such individuals were not breastfed (or if a neonate, they were breastfed for such a short time it is unlikely to be evident in their isotopic values). Method 1 results in slightly fewer fetal/neonate individuals (n = 3) being interpreted as breastfeeding or weaning compared to method 2 (n = 4). As well, method 1 classifies three 1-11-month-olds as breastfeeding (and 2 as weaning) while method 2 does not classify any of these infants as breastfeeding (it does have 2 individuals as weaning although only one of these is the same as in method 1). This suggests method 1 may be more appropriate for detecting breastfeeding and weaning in this sample. The use of isotopic covariation may not be well suited to our data as we have only two data points (the metaphysis and diaphysis) for each individual.

**Table 3:** δ^15^N and δ^13^C results for Beemster nonadults ≤6 years of age with results of feeding status at death criteria. N Bf = no breastfeeding (hence no weaning); Bf = breastfeeding; W = weaning; I = indeterminate (see Figs 2 and 3). Different results between interpretive method 1 and 2 are italicized.

ID Number	Osteological Age	Archival Age	Age cat-egory	Archival ID	Arch-ival Sex	Year of Death	δ^13^C (‰)		Maximum δ^13^C dia-meta difference	δ^15^N (‰)		Maximum δ^15^N dia-meta difference	Coupled Isotope Change & Ratio%	Feeding Status Method 1	Feeding Status Method 2
							meta	dia		meta	dia				
**FETAL & NEONATE (<1 month)**															
S351V1500	31w±1w	-	fetus	-	-	-	-18.1	-19.2	+1.1	14.2	14.0	+0.2	y+ (0.2)	--	--
S90V0107	32w±4w	-	fetus	-	-	-	-20.2	-20.0	-0.2	14.1	14.3	-0.2	y- (1.0)	--	--
S274V0480	34w±2w	-	fetus	-	-	-	-20.0	-19.9	-0.1	14.8	14.4	+0.4	n	--	--
S344V0765	34w±2w	-	fetus	- (found in pelvic cavity of S344 V0730, 20y, F)	-	1836	-21.3	-21.5	+0.2	13.7	13.2	+0.5	y+ (2.5)	*--*	*--* ‡
S102V0151	36w±3w	-	fetus	-	-	-	-20.0	-19.8	-0.2	14.4	15.3	-0.9	y- (4.5)	--†	--†
S138V0499	38w±2w	-	neonate	-	-	-	-21.0	-20.7	-0.3	14.6	15.3	-0.7	y- (2.3)	--†	--†
S139V0215	38w±2w	-	neonate	-	-	-	-20.4	-20.2	-0.2	15.2	15.6	-0.4	y- (2.0)	*--* ‡	*--*
S376V0900	38w±10w	-	neonate	-	-	-	-21.1	-21.0	-0.1	13.9	13.8	+0.1	n	--	--
S230V0302	39w±3w	-	neonate	-	-	-	-20.2	-20.2	0.0	13.9	14.3	-0.4	n	--	--
S418V0906	40w±4w	-	neonate	-	-	-	-19.0	-19.7	+0.7	13.9	13.9	0.0	n	--	--
S274V0418	40w±3w	-	neonate	-	-	-	--	-20.3	n/a	--	14.7	n/a	n/a	--	--
S232V0307	40w±4w	-	neonate	-	-	-	-20.8	-20.8	0.0	14.3	14.4	-0.1	n	--	--
S72V0001	40w±4w	stillborn	neonate	no name Vi.	-	1834	-19.4	-19.9	+0.5	11.8	12.2	-0.4	n	--	--
S320V0662	40w±4w	stillborn	neonate	no name de G.	-	1849	-20.7	-20.5	-0.2	13.5	14.1	-0.6	y- (3.0)	*--*	*--* †
S191V0374	39w±3w	stillborn	neonate	no name Ol.	-	1864	-20.5	-20.4	-0.1	13.3	13.3	0.0	n	--	--
S295V0485	40w±2w	stillborn	neonate	no name O.	-	1840	-20.8	-20.5	-0.3	14.7	14.9	-0.2	y- (0.7)	--	--
S296V0486	39w±4w	stillborn	neonate	no name O.	-	1839	-20.8	-20.6	-0.2	15.0	14.6	+0.4	n	--	--
S164V0364		2d	neonate	Johan v D.	M	1853	-19.4	-19.7	+0.3	14.4	14.5	-0.1	n	--	--
S406V0884		3d	neonate	Trintje O.	F	1834	-20.5	-20.4	-0.1	14.4	14.2	+0.2	n	--	--
S245V0390		3d	neonate	Jannetje M.	F	1848	-20.2	-20.2	0.0	14.5	14.5	0.0	n	--	--
S323V0650		6d	neonate	Grietje v Ei.	F	1840	-20.3	-20.7	+0.4	13.1	13.6	-0.5	n	--	--
S227V0297		6d	neonate	Sientje L. (§)	F	1857	-20.4	-20.5	+0.1	13.9	13.7	+0.2	y+ (2.5)	--	--
S373V0798		7d	neonate	Neeltje Me.	F	1841	-20.6	-21.5	+0.9	14.1	14.1	0.0	n	--	--
S315V0656		14d	neonate	Antje de Bo.	F	1838	-20.7	-20.7	0.0	12.8	13.1	-0.3	n	--	--
S82V0084		17d	neonate	Maartje B.	F	1838	--	-21.1	n/a	--	14.3	n/a	n/a	--	--
S50V0042		19d	neonate	Neeltje Be.	F	1846	-19.3	-20.6	+1.3	14.2	14.1	+0.1	y+ (0.8)	--	--
S0V1524	3w±3w	-	neonate	-	-	-	-20.3	-20.5	+0.2	12.7	13.5	-0.8	n	--	--
**n**							**25**	**27**	**25**	**25**	**27**	**25**	**4 y+;** **6 y-;** **15 n**	**2** † **1** ‡	**3** † **1** ‡
**Mean**							**-20.2**	**-20.4**	**0.15**	**14.0**	**14.1**	**-0.14**			
**SD**							**0.73**	**0.53**	**0.44**	**0.78**	**0.73**	**0.38**			
**YOUNG INFANT (1–11 months)**															
S335V0711		29d	infant	Leonora S. Su.	F	1831	-20.1	-20.1	0.0	15.0	16.1	-1.1	n	*W*	*N Bf*
S400V0859		29d	infant	Cornelis W.	M	-	-20.5	-20.4	-0.1	14.5	14.7	-0.2	y- (2.5)	N Bf	N Bf
S0V0358	1m±1m	-	infant	-	-	-	-20.8	-20.2	-0.6	14.2	15.8	-1.6	y- (2.7)	W	W
S130V0173	1m±1m	-	infant	-	-	-	-20.2	-20.1	-0.1	15.2	15.3	-0.1	y- (1.0)	*Bf*	*N Bf*
S187V0267	1m±3w	-	infant	-	-	-	-21.1	-21.1	0.0	13.6	13.3	+0.3	n	N Bf	N Bf
S273V0619	1m±2w	-	infant	-	-	-	-19.9	-20.0	+0.1	14.0	14.4	-0.4	n	N Bf	N Bf
S287V0450	5w±4w	-	infant	-	-	-	-20.9	-21.1	+0.2	14.3	14.4	-0.1	n	N Bf	N Bf
S99V0139		7w	infant	Jantje vd M.	F	1850	-16.0	-18.4	+2.4	13.0	14.2	-1.2	n	N Bf	N Bf
S133V0299	7w±4w	-	infant	-	-	-	-19.3	-20.0	+0.7	14.1	14.5	-0.4	n	N Bf	N Bf
S352V0747		7w	infant	Catharina B. (§)	F	1845	-19.4	-20.2	+0.8	14.8	14.3	+0.5	y+ (0.6)	N Bf	N Bf
S493V1069	2m±1m	-	infant	-	-	-	-20.9	-21.0	+0.1	13.6	14.3	-0.7	n	N Bf	N Bf
S214V0227	2m±2.5m	-	infant	-	-	-	-19.1	-19.9	+0.8	14.4	14.0	+0.4	y+ (0.5)	N Bf	N Bf
S330V0706	2.5m±2.5m	-	infant	-	-	-	--	-19.6	n/a	--	14.6	n/a	n/a	--	--
S314V0655		10w	infant	Teunis de Bo(§)	M	1845	-19.8	-19.8	0.0	11.9	12.6	-0.7	n	N Bf	N Bf
S152V0244		11w	infant	Pieter E.	M	1861	-20.0	-20.0	0.0	15.4	15.7	-0.3	n	*Bf*	*N Bf*
S37V0021		3m	infant	Cornelius T.	M	1858	-19.7	-20.5	+0.8	13.8	14.5	-0.7	n	N Bf	N Bf
S421V0940	5m±2m	-	infant	-	-	-	--	-19.7	n/a	--	15.4	n/a	n/a	--	--
S122V0161	5m±2.5w	-	infant	-	-	-	-18.9	-18.3	-0.6	14.4	13.8	+0.6	n	N Bf	N Bf
S190V0310		5m	infant	Klaas de Bo. (§)	M	1844	-18.8	-18.8	0.0	14.8	14.4	+0.4	n	N Bf	N Bf
S86V0106	6m±3m	-	infant	-	-	-	-19.1	-19.2	+0.1	16.6	15.3	+1.3	y+ (13.0)	*Bf*	*N Bf*
S80V0049	7m±2m	-	infant	-	-	-	-19.3	-19.5	+0.2	12.8	13.5	-0.7	n	N Bf	N Bf
S91V0110		7 or 8m	infant	no name (§)	M	1856	--	-18.7	n/a	--	12.9	n/a	n/a	--	--
S103V0153	8m+/-6m	-	infant	-	-	-	--	-18.5	n/a	--	12.8	n/a	n/a	--	--
S241V0359	9m±3m	-	infant	-	-	-	-21.5	-21.1	-0.4	13.2	13.8	-0.6	y- (1.5)	*N Bf*	*W*
**n**							**20**	**23**	**20**	**20**	**23**	**20**	**3 y+;** **4 y-;** **13 n**	**3 Bf;** **2 W;** **15 N Bf;** **0 I**	**0 Bf;** **2 W;** **18 N Bf**
**Mean**							**-19.8**	**-19.8**	**0.22**	**14.2**	**14.3**	**-0.27**			
** SD**							**1.18**	**0.89**	**0.66**	**1.03**	**0.94**	**0.64**			
**OLDER INFANT (1 year)**															
S231V0305	15m±3m	-	infant	-	-		--	-20.8	n/a	--	14.1	n/a	n/a	--	--
S58V0092		17m	infant	Henri T. Th.	M	1842	-18.5	-18.0	-0.5	13.1	13.8	-0.7	y- (1.4)	*N Bf*	*W*
S106V0142	1.75y±6m	-	infant	-	-	-	-20.5	-20.3	-0.2	13.2	12.9	+0.3	n	N Bf	N Bf
S38V0026		22m	infant	Klaas vd M.	M	1850	-21.4	-21.4	0.0	12.8	13.3	-0.5	n	N Bf	N Bf
S127V0204		23m	infant	Cornelia v L (§)	F	1842	-20.1	-20.0	-0.1	13.7	13.5	+0.2	n	N Bf	N Bf
**n**							**4**	**5**	**4**	**4**	**5**	**4**	**0 y+;** **1 y-;** **3 n**	**0 Bf;** **0 W;** **4 N Bf;** **0 I**	**0 Bf;** **1 W;** **3 N Bf**
** Mean**							**-20.1**	**-20.1**	**0.20**	**13.2**	**13.5**	**-0.18**			
** SD**							**1.21**	**1.29**	**0.22**	**0.37**	**0.46**	**0.50**			
**TODDLER (2 years)**															
S24V0086	2y±1y	-	infant	-	-	-	-19.5	-19.3	-0.2	13.5	13.5	0.0	n	N Bf	N Bf
S158V0230		24m	infant	Trijntje M.	F	1861	-21.1	-20.5	-0.6	12.9	12.7	+0.2	n	N Bf	N Bf
S35V0031		2y	infant	Adrianus Kl.	M	1846	-20.7	-20.7	0.0	13.9	13.4	+0.5	n	N Bf	N Bf
S165V0242		24m	infant	Maartje Sm.	F	1857	-21.3	-21.0	-0.3	14.3	14.7	-0.4	y- (1.3)	N Bf	N Bf
S328V0701		28m	infant	Trijntje K.	F	1832	-21.1	-20.6	-0.5	14.5	14.9	-0.4	y- (0.8)	*I*	*N Bf*
S318V0643		30m	infant	Dirk R. (§)	M	1845	-20.8	-21.8	+1.0	12.6	13.4	-0.8	n	N Bf	N Bf
S44V0020	2.5y±1y	-	infant	-	-	-	-20.4	-20.6	+0.2	13.1	13.3	-0.2	n	N Bf	N Bf
S284V0466	2.5y±1y	-	infant	-	-	-	-19.5	-20.3	+0.8	14.2	13.2	+1.0	y+ (1.3)	N Bf	N Bf
S46V0023		32m	infant	Trintje Bs. (§)	F	1842	-21.0	-20.9	-0.1	13.8	15.0	-1.2	y- (12.0)	*W*	*N Bf*
**n**							**9**	**9**	**9**	**9**	**9**	**9**	**1 y+;** **3 y-;** **5 n**	**0 Bf;** **1 W;** **7 N Bf;** **1 I**	**0 Bf;** **0 W;** **9 N Bf**
**Mean**							**-20.6**	**-20.6**	**0.03**	**13.6**	**13.8**	**-0.14**			
** SD**							**0.68**	**0.66**	**0.55**	**0.66**	**0.84**	**0.67**			
**CHILD (3–6 years)**															
S62V0071		3y	child	no name	M	1842	-20.6	-21.1	+0.5	14.7	15.5	-0.7	n	*I*	*N Bf*
S145V0238		3y0m	child	Dirk S. (§)	M	1862	-19.3	-20.1	+0.8	12.2	12.7	-0.4	n	N Bf	N Bf
S89V0091		3y1m	child	Trintje Bo.	F	1832	-21.6	-21.5	-0.1	14.9	15.9	-1.0	y- (10.0)	*W*	*N Bf*
S252V0443		3y4m	child	Frederika Catharine Ol.	F	1849	-20.2	-19.9	-0.3	13.7	14.0	-0.3	y- (1.0)	N Bf	N Bf
S343V0732		3y5m	child	Cornelis M.	M	1855	-21.2	--	n/a	13.2	--	n/a	n/a	--	--
S177V0413		3y6m	child	Geertje L.	F	1863	-19.7	-19.7	0.0	14.4	14.2	+0.2	n	N Bf	N Bf
S255V0398	3y±1y	-	child	-	-	-	-21.1	-20.6	-0.5	11.9	13.0	-1.1	y- (2.2)	*N Bf*	*W*
S293V0495	3y±1y		child		M		-20.6	-20.1	-0.5	13.8	14.9	-1.1	y- (2.2)	W	W
S316V0641		3y7m	child	Klaas Bi. (§)	M	1839	-20.7	-20.7	0.0	12.6	12.8	-0.2	n	N Bf	N Bf
S189V0332	3.5y±1y	-	child	-	-	-	-17.7	-20.3	+2.6	13.9	13.9	0.0	n	N Bf	N Bf
S32V0082	3.5y±1y	-	child	-	-	-	-21.4	-21.2	-0.2	13.6	12.9	+0.7	n	N Bf	N Bf
S141V0223	3.5y±1y	-	child	-	-	-	-20.7	-20.8	+0.1	14.8	14.6	+0.2	y+ (2.0)	N Bf	N Bf
S326V0708		3y11m	child	Grietja H. (§)	F	1836	-20.9	-21.0	+0.2	12.3	13.0	-0.7	n	N Bf	N Bf
S351V0756		3y11m	child	Cornelius B. (§)	M	1847	-20.5	-20.5	0.0	12.5	12.9	-0.4	n	N Bf	N Bf
S463V0988	4y±1y	-	child	-	-	-	-21.1	-20.5	-0.6	13.5	13.3	+0.2	n	N Bf	N Bf
S109V0175	4y±1y	-	child	-	-	-	-20.6	-20.5	-0.1	13.5	12.9	+0.6	n	N Bf	N Bf
S105V0170	4y±1y	-	child	-	-	-	-20.6	-20.7	+0.1	13.4	13.1	+0.3	y+ (3.0)	N Bf	N Bf
S104V0136		4y7m	child	Jan Sch.	M	1857	-20.6	-21.0	+0.4	13.1	13.1	0.0	n	N Bf	N Bf
S181V0407		4y7m	child	Arie Bi.	M	1839	-21.1	-21.4	+0.3	12.5	12.9	-0.4	n	N Bf	N Bf
S215V0250		4y10m	child	Aaltje d G.	F	1851	-20.9	-20.6	-0.3	13.8	14.1	-0.3	y- (1.0)	N Bf	N Bf
S353V0739	4.5y±1y	-	child	-	-	-	-20.5	-20.5	0.0	13.3	14.5	-1.2	n	*W*	*N Bf*
S336V0709		5y3m	child	Aaltje Br.	F	1847	-20.5	-20.9	+0.4	13.6	13.7	-0.1	n	N Bf	N Bf
S140V0207		5y6m	child	Gerrit d G.	M	1859	-20.1	-20.5	+0.4	14.0	13.9	+0.1	y+ (0.3)	N Bf	N Bf
**n**							**23**	**22**	**22**	**23**	**22**	**22**	**3 y+;** **5 y-;** **14 n**	**0 Bf;** **3 W;** **18 N Bf; 1 I**	**0 Bf;** **2 W;** **20 N Bf**
**Mean**							**-20.5**	**-20.6**	**0.15**	**13.4**	**13.7**	**-0.25**			
**SD**							**0.80**	**0.46**	**0.65**	**0.83**	**0.92**	**0.54**			

meta = metaphysis, dia = diaphysis; d = days, w = weeks, y = years, M = male, F = female.

† = fetus with a pattern of ẟ^15^N that would be interpreted as weaning.

‡ = fetus with a pattern of ẟ^15^N that would be interpreted as breastfeeding.

§ = indicates a probable but not certain archival match.

In interpretive method 1, the 1-11-month-olds (young infants) have five out of 20 individuals (25%) with evidence of being breastfed (3 categorized as breastfeeding and 2 as weaning). The individuals classified as ‘weaning’ are very young, both around 1-month of age. The individuals categorized as ‘breastfeeding’ are 1.0±1.0 month, 11 weeks, and 6.0±3.0 months of age. The other 15 individuals (75%) lack isotopic evidence suggestive of breastfeeding or weaning, being thus classified as ‘no breastfeeding’. In interpretive method 2, none of the 1-11-month-olds are classified as breastfeeding, two (10%) are classified as weaning, and the remaining 18 (90%) are thus denoted as ‘no breastfeeding’.

In interpretive method 1, none of the 1-year-olds (older infants) have isotopic evidence for having been breastfed, although the sample size is small (n = 4). In interpretive method 2, one (25%) of the 1-year-olds is classified as weaning while the other three (75%) are classified as ‘no breastfeeding’. In method 1, the 2-year-olds (toddlers) have seven of nine individuals (77.8%) who lack isotopic evidence of having been breastfed, with one individual (11.1%) categorized as ‘weaning’ and another as ‘indeterminate’ (11.1%). In method 2, all nine 2-year-olds are classified as ‘no breastfeeding’. Finally, in the 3-6-year-olds (children), method 1 results in 18 out of 22 individuals (81.8%) being categorized as ‘no breastfeeding’, while three (13.6%) are categorized as ‘weaning’ and one (4.5%) is ‘indeterminate’. In method 2, two individuals (9.1%) are classified as weaning and the remaining 20 (90.9%) are classified as ‘no breastfeeding’. In all individuals, elevated ẟ^15^N values consistent with breastfeeding or weaning could instead be due to other causes of ^15^N enrichment (i.e. protein catabolism from malnutrition or wasting disease; high trophic-level foods). Given the short to absent period of breastfeeding in the infants, alternative causes of ^15^N enrichment are quite likely in the toddlers and children.

**Fig 5:**
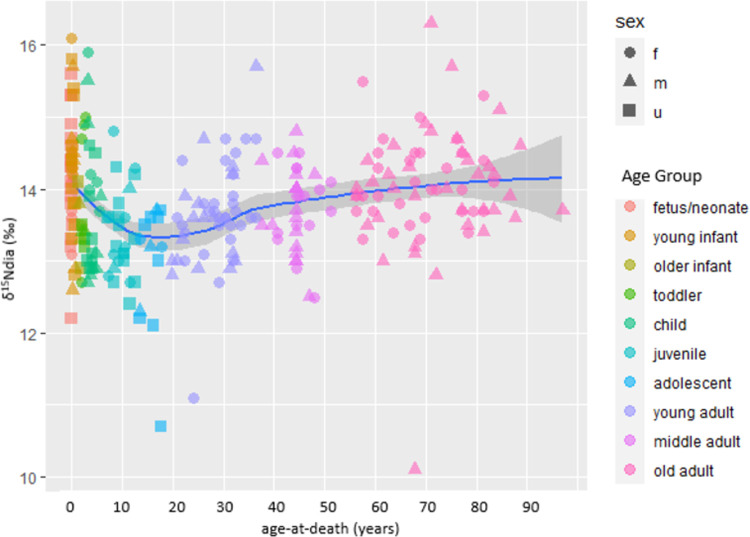
δ^15^N values of nonadults (diaphyseal values) and adults from Beemster. The blue line is the best-fit smoothed (local) regression line (using loess algorithm) and the grey bands are the 95-percent confidence interval.

**Fig 6:**
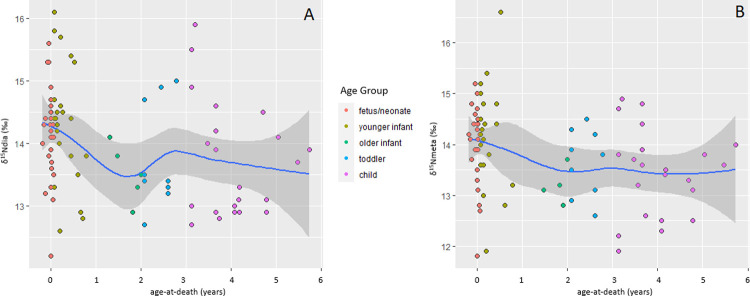
δ^15^N values of ≤6-year-olds from Beemster. A) Diaphyseal values, B) metaphyseal values. The blue line is the best-fit smoothed (local) regression line (using loess algorithm) and the grey bands are the 95-percent confidence interval.

Fig 5 plots the ẟ^15^N values of all nonadults (using diaphyseal values) and adults. Fig 6 plots the diaphyseal and metaphyseal ẟ^15^N values of the ≤6-year-olds so the isotopic variation can be seen more clearly. The fetuses/neonates have a spread of ẟ^15^N values of 3.8‰ which closely corresponds to the spread of reproductively-aged Fs at 3.6‰. The young infants (1–11 months) have the largest spread of ẟ^15^N values, at 4.7‰, suggesting considerable variation in diet and physiology, some of which may be due to breastfeeding (even if of short duration). Yet, the breastfeeding signal is minor, nowhere near the 2 to 3‰ we might expect, as the young infant mean (14.3‰) is only 0.2‰ higher and not statistically different than the fetal/neonate mean (14.3‰; t = 0.732, p = 0.468) and only 0.6‰ higher than the mean of the Fs of reproductive age (13.7‰); this latter difference is statistically significant (t = 3.227, p = 0.002; the fetal-neonate and reproductively-aged F means also differ significantly, t = 2.711; p = 0.008). By 1 year-of-age ẟ^15^N values means have dropped such that sample-wide interpretations suggest the absence of breastfeeding although individually-based interpretations show a few individuals with isotopic patterning that could be consistent with continued breastmilk consumption (but other factors could be the cause). The older infants and toddlers have rather small ẟ^15^N spreads (1.3 and 2.4‰, respectively) which is likely in part related to the smaller sample sizes of these groups. The larger sample of children has a correspondingly larger spread of ẟ^15^N values at 4.0‰, and with a ẟ^15^N mean of 13.7‰, suggests that even in the absence of breastfeeding, dietary and physiological variation can cause considerable isotopic variation. Finally, the juveniles (7–12 years) and adolescents (13–17 years) have rather low ẟ^15^N means, at respectively, 13.4‰, and then the lowest of all, 12.9‰, with moderate ẟ^15^N spreads of 2.4‰ and 3.0‰.

No significant ẟ^15^N differences are found between archivally identified nonadult M and F age groups, but sample sizes are often small (S3 Table). To generate larger sample sizes, all 1-month to 6-year-olds with an archival sex identification are compared (M = 18; F = 14): no statistical differences are present (metaphysis: t = 1.516, p = 0.140; diaphysis: t = 1.615, p = 0.117) nor are differences precent when comparing all post-neonate nonadults (using the diaphyseal values of the young infants to children) (M = 23, $\bar{x}$ = 13.6‰, SD = 0.94‰; F = 25, $\bar{x}$ = 13.9‰, SD = 0.94‰; t = 1.054, p = 0.297).

**Fig 7:**
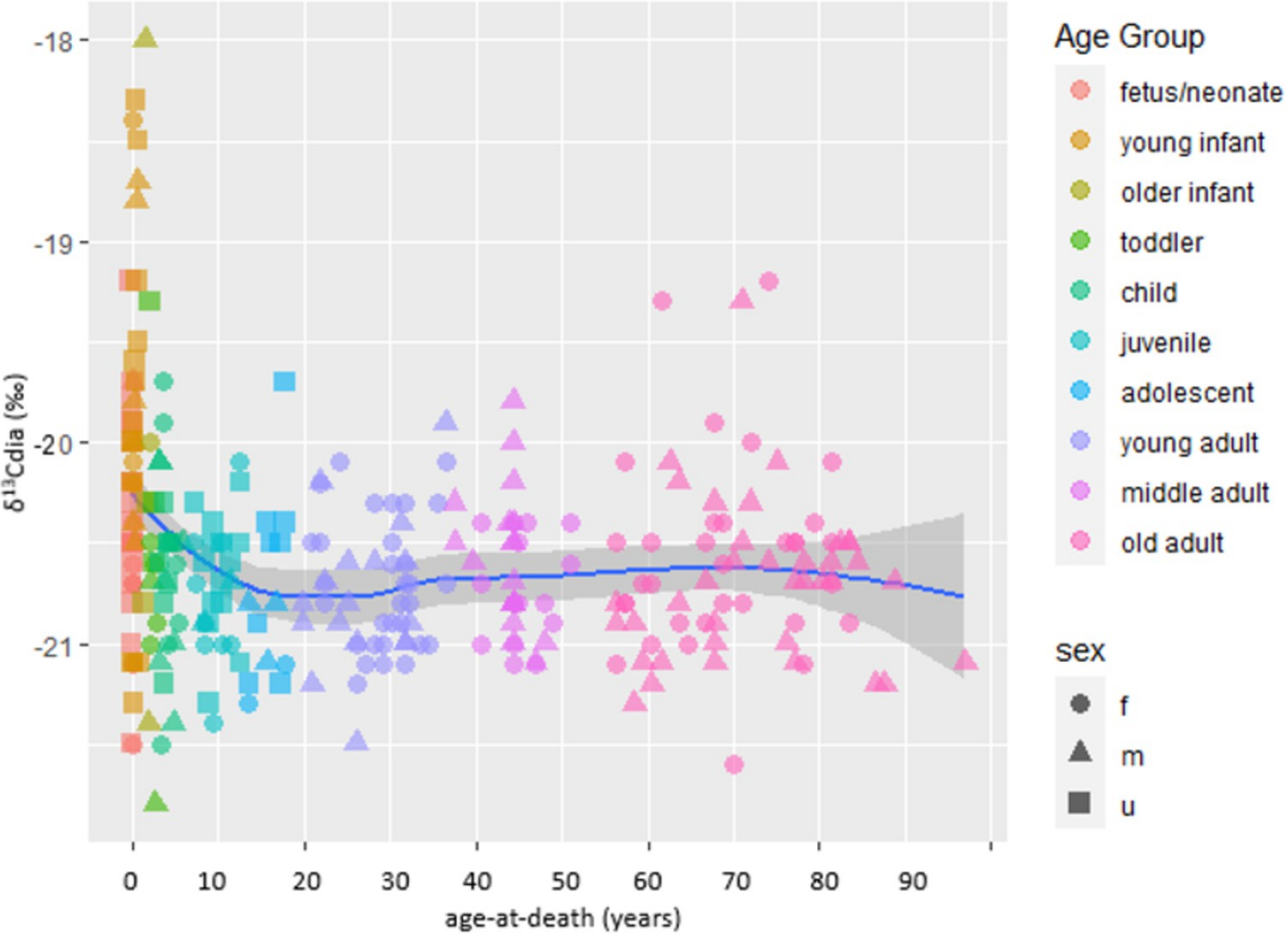
δ^13^C values of nonadults (diaphyseal values) and adults from Beemster. The blue line is the best-fit smoothed (local) regression line (using loess algorithm) and the grey bands are the 95-percent confidence interval.

**Fig 8:**
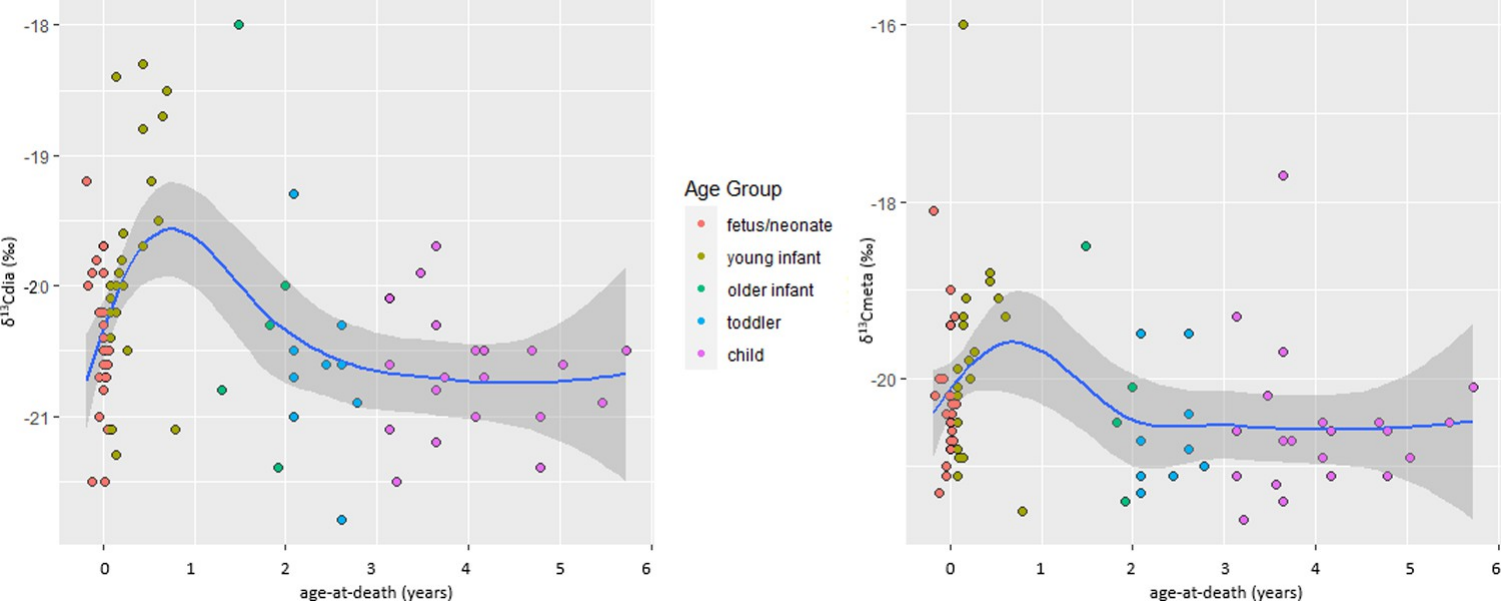
δ^13^C values of ≤6-year-olds from Beemster. A) Diaphyseal values, B) metaphyseal values. The blue line is the best-fit smoothed (local) regression line (using loess algorithm) and the grey bands are the 95-percent confidence interval.

Fig 7 plots the ẟ^13^C values of all nonadults (using diaphyseal values) and adults while Fig 8 plots the diaphyseal and metaphyseal ẟ^13^C values of the ≤6-year-olds. Like the ẟ^15^N data, the young infants (1–11 months) have the largest spread of ẟ^13^C values at 5.5‰ and the highest ẟ^13^C mean of -19.8‰. This compares to a fetal-neonate ẟ^13^C spread of 3.4‰ and significantly different mean of -20.4‰ (U = 182.000, p = 0.012) and a reproductively-aged F ẟ^13^C spread of only 1.1‰ and significantly different mean of -20.7‰ (U = 208.000; p = 0.000; the fetal-neonate group also differs significantly from the reproductively aged F group, U = 381.500, p = 0.003). The older infants (1 year) have the next highest ẟ^13^C mean at -20.2‰ with a spread of 3.4‰. The remaining nonadult and adult age groups have similar ẟ^13^C means, of -20.6 to -20.8‰ and maximum to minimum spreads of 3.9‰ (child) to 0.7‰ (MA F). Thus, the ẟ^13^C data support the suggestion that a short period of breastfeeding in some young infants may be contributing to their high isotopic variation, but fairly high levels of ẟ^13^C variation are present in other age groups and, as will be discussed, special weaning foods may be contributing to the young infant results.

No significant ẟ^13^C differences are found between archivally identified nonadult M and F age groups (S3 Table). The statistical comparison of combined groups with larger sample sizes yielded no significant differences (all 1-month to 6-year-olds: metaphysis: t = -0.016, p = 0.987, diaphysis: t = -0.228, p = 0.821; all post-neonate nonadults (using the diaphyseal values of the young infants to children): t = -0.729, p = 0.470).

The final set of statistical assessments involve investigation of temporal trends for those individuals with archival data specifying year of death. There are no significant correlations between year of death and ẟ^15^N or ẟ^13^C for any age or age-sex group (S3 Table).

## Discussion

### Beemster diet

The isotope data are consistent with historical information about the average western Dutch diet, supporting that the staples consisted of C3 grains and vegetables and, less commonly, animal protein from domesticated fowl/eggs and terrestrial herbivores. Some of the domesticated herbivore isotope values are higher than might be expected (between 8–10‰), which is likely due to a ‘manuring effect’ [102]. We know dairy products were a staple, but these have isotopic values in the range of the cattle, so it is not possible to distinguish how much protein was derived from dairy vs. meat/eggs. Marine foods seem to have been rarely consumed in 19^th^ century Beemster, in keeping with suggestions from a modern-day Beemster historian (Visser, personal communication).

Overall the diet appears to have been fairly homogeneous, shown by temporal continuity in adult and nonadult isotope values, the high similarity of M and F isotope data (i.e., in all the adults and nonadults with archival identification), and the moderate similarity between nonadult and adult diet. The isotopic differences among the means of the different age groups are fairly small (maximum 1.4‰ for ẟ^15^N and 1.0‰ for ẟ^13^C) even including the young infant age group which often has the greatest isotopic dissimilarity because of breastfeeding. For the mean ẟ^15^N results, it is the adolescents that vary the most, with a low value of 12.9‰. The juveniles also have a slightly low δ^15^N mean at 13.4%. Several studies have found similarly low ẟ^15^N values in juveniles/adolescents [see 3], suggesting a common physiological event such as growth may the cause. However, research thus far has found limited support for this explanation so it may be that this age group often consumed foods from lower trophic levels [3, 103, 104]. Amongst the overall population-wide isotopic homogeneity, there is variation amongst the nonadults that is now discussed as it relates to infant feeding practices.

### Infant feeding practices

Interpreting the nonadult isotope data is challenging because infant bone collagen values reflect multiple sometimes co-occurring variables, specifically fetal metabolism, maternal diet, breastfeeding, complimentary feeding, and infant and mother physiology and health, that are difficult (even impossible) to completely disentangle [79]. This is clear in Figs 5–8 which show that the young infants have the most isotopic heterogeneity. Viewed cross-sectionally, the slightly higher δ^15^N and δ^13^C means of the young infants probably suggest some individuals were breastfed, for at least a short period. Even with metaphyseal samples meant to capture a shorter period of time than other bones, the time-averaging may be too great to clearly pick up a short breastfeeding period, especially if the δ^15^N tropic shift is less pronounced because complimentary feeding began at a very young age meaning breastfeeding episodes were less frequent and non-maternal foods made up a significant portion of the diet. This is a large part of why this intra-long bone approach seemingly worked better in a population with a long breastfeeding-weaning period of 3–4 years [89, 105, 106]. δ^15^N values reflect dietary protein, and, if soon after birth, the predominate protein source was cow’s milk we would not see markedly higher infant values. Breastfeeding could conceivably have continued for longer than suggested by the bone collagen data especially if feeding episodes were infrequent and/or of short duration.

Yet, whether looking cross-sectionally or at the individual data (i.e. categorization of feeding status at death), and whether using interpretive method 1 or 2, the isotopic data suggest that most infants were either not breastfed or only breastfed for a short period (weeks to a few months). Depending on the interpretive method, only two to five out of twenty young infants (10–25%) have isotopic evidence of having been breastfed while the remainder lack such patterning. There are no clear age differences between young infants with and without isotopic evidence of breastfeeding, as both contain 1 to 6-month-olds. While there are a few individuals in the toddler and child categories with δ^15^N values that could be due to breastfeeding, given the short or possibly absent period of breastfeeding in the infants, it is more likely these individuals have elevated δ^15^N values because of consumption of high trophic foods, malnutrition or a wasting disease.

Three (12%) or four (16%) Beemster fetuses/neonates, depending on which interpretive method is used, have δ^15^N values that would be interpreted as suggesting breastfeeding or weaning if they were in the infant category. If these individuals are indeed fetuses/neonates (none have archival identification but age-estimation was based on dental formation standards [107, 108] which is the most accurate method available so the majority are probably not infants), they were obviously not breastfed and their elevated ẟ^15^N values are likely due to (a) maternal or infant physiological stress during the gestational period, (b) fetal amino acid metabolism or placental isotope fractionation, or (c) maternal dietary change (consumption of more ^15^N-enriched foods). It is unlikely Beemster women consumed a special pregnancy diet, given the similarity of adult isotope values across the sexes and age-groups (granted these values derive from bone collagen which reflects a long time-averaged signal that would miss short-term dietary changes) and a lack of historical documents that mention any such practice. A common 17^th^ and 18^th^ century household Dutch medical manual warned expectant mothers about the ills of eating hot, cold, and spicy foods and to ensure proper nutrition, but specific foods are not detailed [109]. Given the post-Medieval Dutch dietary staples, common cooking techniques (e.g., large pots of once heated ‘stews’ eaten throughout the day), and the obvious absence of refrigeration and temperate climate, this would seemingly apply to few foods. Nineteenth century texts only mention the importance of good nutrition during pregnancy without further detail [28, 30, 110]. Thus, while we cannot rule out maternal dietary changes as the cause of high fetal ẟ^15^N values the more likely explanation lies in physiological processes.

Other bioarchaeology studies have found high fetal/perinate ẟ^15^N values (relative to adult females) [100, 111] and research on modern mother-infant dyads suggests a non-dietary cause related to fetal amino acid metabolism, placental fractionation, and/or maternal nutritional stress [58, 112–114] [also see 115]. The latter seems slight or uncommon in this sample because nutritional stress causes maternal fat stores, depleted in ^13^C relative to proteins and carbohydrates, to be used by the fetus causing lower fetal ẟ^13^C values [76, 116] which is absent here (the fetal ẟ^13^C mean is 0.3‰ higher than the reproductively-aged Fs which is similar to the 0.4‰ difference in modern paired fetal-maternal samples observed by [112]). Thus, the explanation for elevated fetal/neonate δ^15^N values is most consistent with fetal physiological processes.

The ẟ^13^C data shed light on the nature of the weaning foods. Interestingly, the ẟ^13^C by age results (Fig 8) are a better example of the ‘curve’ we expect to see with breastfeeding than the ẟ^15^N by age results (Fig 6), due to young infants having values that are 0.6‰ and 0.9‰, respectively, above the fetal/neonate and reproductively-aged F means. Indeed, our ẟ^13^C results are consistent with the roughly 1‰ ẟ^13^C breastfeeding trophic shift and may suggest breastfeeding was more common/prolonged than suggested by the ẟ^15^N data. A similar pattern was observed by Craig-Atkins et al. [1], who argued that the absence of a rise in infant δ^15^N values accompanied by a peak in δ^13^C values suggested limited breastfeeding alongside supplementary foods with less negative δ^13^C values than maternal diet, and Beaumont et al. [100], who suggested ẟ^13^C values were less affected by physiological changes than ẟ^15^N values so provided a more robust measure of breastfeeding and weaning. We also suggest that the enriched ẟ^13^C values are most likely due to the isotopic composition of weaning foods, which the ẟ^15^N results and historical information suggest were offered at a very young age. Several historical texts mention that sugar was added to paps and animal milks and some of this would have come from the C4 sugarcane plant with elevated ẟ^13^C values [28, 29, 117, 118]. For example, “In 1876 the infant food in Friesland was, as a rule, mother’s milk…It was the habit, in addition to breast milk, to give the infant very soon [after birth] milk from animals (cows, sheep, goats), either diluted with water alone or as a buttermilk with buckwheat flour and sugar or syrup, or a porridge (of white bread, rusk, buckwheat groats with diluted milk, butter and sugar)"[16: 10, 11, translation ours]. Sugarcane had become popular in post-Medieval continental Europe. While it was unavailable in the early 1800s as a result of Napoleon’s Continental Blockade (closing European markets to British ships and goods, circa 1806–1814), which spurred the development of a new sugar beet industry, by the ~1830s the Dutch sugarcane industry had recovered such that Amsterdam became Europe’s chief ‘sugar town’ with both cane and beet varieties available [20, 22]. While much of the product was for export, Dutch consumption of sugar increased in tandem with its decreasing cost over the rest of the century [20, 119]. Many Beemster farmers could likely afford sugar, or a syrup made from it, given their advantageous economic position. Previous isotope studies have considered higher ẟ^13^C values in infants as a result of weaning foods containing maize and millet (or animals consuming these plants) [59, 120–122] but sugarcane has not yet been seriously considered.

#### Why was breastfeeding short to absent in Beemster?

In 17^th^ century Netherlands, breastfeeding purportedly lasted for about two years [123] but we lack data about geographic or temporal variation in this century and the next. By the 19^th^ century there are reports that breastfeeding lasted for a year or two, for example, an 1812 report said most Dutch mothers breastfed for one year but that in North Holland feeding young infants both breastmilk and porridge was especially common [Persman, 1812 in 16]. Other historical texts discuss the common use of pap with or instead of breastmilk in Amsterdam, with pap being given sometimes as early as a few days, and that infants of low SES workers in Utrecht were often not breastfed (in contrast to the wealthy of the city) [22, 124].

To the best of our knowledge there are no historical texts that refer to infant feeding in Beemster specifically, however, many of its characteristics suggested that breastfeeding would have been fairly common and of at least average duration. These are that it was a predominately Protestant community of moderate to high SES, its northern, rural location, and that mothers worked in/near the home. Yet, this is the opposite of what we found. This highlights the importance of studying each region and not assuming these characteristics can be used as predictors of infant feeding practices. For late 19^th^ to early 20^th^ century France, Rollet [125] found the geographical distribution of dairy farming was associated with low rates of breastfeeding so there is precedence for this pattern in one other European nation. These studies suggest dietary differences between geographic regions do affect infant feeding practices, specifically that dairying regions might be more inclined to fed infants animal milks instead of breastmilk and to offer these from a very young age. Future research should study the breastfeeding and weaning practices of other (post-)Medieval dairying communities; perhaps these will reveal populations that were regularly practicing artificial feeding even earlier than the 19^th^ century.

Other European areas with historically reported communities that had high rates of artificial feeding in the post-Medieval period are northern and southern Germany, the Tyrol area of Austria, northern France, western Finland, northern Sweden, and Iceland [118, 125–127]. Excessive workload and cultural values and traditions were the common reasons given by mothers for not breastfeeding [126]. Several Dutch studies have found that, indeed, regional variation actually supersedes all other variables in understanding differential IMRs, which thus implies regional variation in infant feeding practices [32, 35]. The impact of an overarching religious ideology such as Catholicism on infant practices has especially been questioned lately [33, 38, 43]. Women’s clothing that was harmful or inconvenient for breastfeeding has been mentioned as factor in Catholic communities [39] but the traditional dress of 19^th^ century Protestant women from North Holland also had a very tight bodice that may have dissuaded breastfeeding.

North Holland women usually did not state an “occupation” on marriage certificates if their work was subsumed within the business of the male head of house (i.e. father, husband) and/or if they did not receive a separate wage [128, 129]. Thus, despite their extensive work on the farm, census enumerators and household members tended to underreport women’s contributions [129]. Still, historical texts shed some light on the working conditions at North Holland dairy farms [128]. The average workday was 12 to 16 hours with a ~2 hour midday break for the main meal; a half day was worked on Sunday [128]. Women were in charge of milking cows and dairy production, on top of meal preparation, domestic chores and rearing children, certainly leading to long working days [69, 70, 129]. Thus, perhaps an important factor in Beemster’s short-absent breastfeeding period was the many other responsibilities of women. While the mother and infant were not separated in space like mothers working in urban factories, the multitude of time-consuming essential tasks may have limited the time available for infant feeding. Children were expected to start working in the house and farm from about 5 years-of-age [123] and could be tasked with feeding an infant cow’s milk, which was always available, thus freeing up the mother to care for the cattle and produce the dairy products that were a major element of the family’s livelihood.

Our study supports suggestions that too singular a focus on religion, location, or occupation, risks missing other factors important in infant feeding behaviours. On top of illustrating the need for regional characterization this work shows the benefits of combining isotopic data with historical texts. Isotopic data can reveal the limitations of historical literary sources as most are generalized, anecdotally-based, subjective estimates that do not reflect actual practice [7, 130–132]. Yet, in this study, historical sources never suggested there was a difference in how long boys vs. girls were breastfed or weaned, which concurs with our isotopic data. As well, the historical records provided invaluable information to refine our interpretations, for example, that the elevated infant ẟ^13^C values were most likely the result of sugar being added to cow’s milk or pap.

#### Limitations and future research

Ongoing incremental dentine collagen sampling should provide the precision needed to more accurately gauge longitudinal dietary changes, infer the presence or absence of a breastfeeding period, determine the heterogeneity of infant feeding practices, and permit a more detailed assessment of temporal changes. Given the rapid innovation of artificial feeding devices, their lowering cost, and increasing availability in the 19^th^ century, we might have expected to see a temporal change in infant feeding patterns across the nearly four decades of Beemster life. On the other hand, if older siblings were usually available to fed babies cow’s milk then these changes might have been irrelevant for Beemster mothers. Because tooth dentine does not turnover (remodel) once formed, its analysis will also yield important information about the feeding patterns of survivors vs. non-survivors to determine if our current results need revision because of a mortality bias wherein those that survived infancy and childhood were breastfed more frequently or for a longer duration. While we can conclude from these data that breastfeeding was short or even absent in most individuals, we recognize that a population-wide weaning trajectory may not have existed.

Compared to traditional protocols involving the analysis of a single sample per individual, this intra-long bone method proved useful in detecting both the presence and direction of isotopic change in the weeks to months before death. Metaphyseal samples from growing long bones will be less affected by the time-averaging caused by modeling and remodeling of bone collagen in other bones like ribs, so can record shorter-term isotopic changes. Yet, our method assumes that a long bone was somewhat continuously growing during its formation, such that a period of dietary change will be recorded in the newly formed metaphyseal collagen. However, growth is saltatory and especially if an individual experienced a period of malnutrition or disease, bone growth may have ceased such that any co-occurring dietary change would be not recorded [100]. This is another reason for ongoing dentine isotope analyses, as dental formation is less prone to growth interruption meaning it is less likely to miss recording the isotope values of periods of physiological stress [100].

The limitations of using population-level aggregate means to interpret individual feeding patterns (a component of interpretive method 1) have been mentioned and the use of isotopic covariation to distinguish dietary from non-dietary isotopic change (the basis of interpretive method 2) may not work well with only two data points per individual. The suitability of using the adult F aggregate results is strengthened by the large sample size, as the reproductively-aged F δ^15^N mean likely included many pregnant and breastfeeding mothers. As well, the use of a fairly large sample of fetuses and neonates is a plus since they are a more appropriate baseline against which to gauge isotopic variation in the young infants. Yet, the data are still cross-sectional and future longitudinal dietary data from incremental dentine may change our interpretations. There is no doubt that bulk bone collagen isotope data provide a less accurate and detailed infant feeding reconstruction than incremental dentine data [79, 100, 133]. However, we were able to study many more individuals than would have been possible if analyzing multiple dentine samples for every individual. This approach provided a broad overview of population diet and detected a short to possibly absent breastfeeding period that can be investigated in more detail through informed selection of individuals for subsequent incremental dentine analyses. This work also initiated research on demographic variables that are affected by breastfeeding and weaning practices and using the historical archival data we have found support for a short or absent breastfeeding period in the infant mortality rate, birth intervals, and differential seasonal mortality, to be detailed in a forthcoming publication.

## Conclusions

Stable isotope research on a large sample of 19^th^ century rural villagers from Beemster, the Netherlands, highlighted the importance of regional cultures of infant feeding. Despite Beemster having many cultural characteristics associated with high breastfeeding rates of long duration in other parts of the country (i.e., Protestant, rural, northern, moderate-high SES, mothers worked at home) breastfeeding was likely of short duration or not practiced at all. There are many bioarchaeological studies about past populations that had moderate to long breastfeeding periods, as artificial feeding was generally not an option. But as this work shows, there are communities in our historic past that had short to even absent breastfeeding periods that are in need of study [134]. The reasons for artificial feeding and choice of breastmilk substitutes are underexplored topics that are aided by historical texts. The great range of human cultural variation not only includes populations who breastfed for markedly different lengths of time, but also past populations that did not breastfed whose investigation will demonstrate the multitude of complex biological, environmental, socio-economic and ideological factors that influenced how a mother decided to feed her baby.

## Supporting information

S1 Table Faunal isotope values used in Fig 4. (XLSX)

S2 Table Sample, isotope and preservation results for Beemster individuals. (DOCX)

S3 Table Results of statistical tests not specified in main paper. (DOCX)
