# Supplementary material for: Isotopic reconstruction of short to absent breastfeeding in a 19th century rural Dutch community
Source: PLoS One. 2022 Apr 13;17(4):e0265821. doi: 10.1371/journal.pone.0265821 (PMC9007374; doi:10.1371/journal.pone.0265821)
Supplement: S2 Table — (DOCX) [file pone.0265821.s002.docx]

Supplement Table 2: Sample, isotope and collagen preservation results for Beemster individuals.

| ID Number | Bone Analysed | Metaphysis Samples | | | | | | Diaphysis Samples | | | | | |
| --- | --- | --- | --- | --- | --- | --- | --- | --- | --- | --- | --- | --- | --- |
|  |  | δ^13^C (‰) | δ^15^N (‰) | %N by weight | %C by weight | C/N ratio | collagen yield^1^ | δ^13^C (‰) | δ^15^N (‰) | %N by weight | %C by weight | C/N ratio | collagen yield^1^ |
| S351V1500 | Humerus | -18.1 | 14.2 | 16.7 | 46.4 | 3.2 | 7.9 | -19.2 | 14.0 | 16.4 | 45.8 | 3.3 | 11.0 |
| S90V0107 | Humerus | -20.2 | 14.1 | 16.1 | 44.9 | 3.2 | error | -20.0 | 14.3 | 16.3 | 44.8 | 3.2 | error |
| S274V0480 | Humerus | -20.0 | 14.8 | 16.3 | 45.0 | 3.2 | error | -19.9 | 14.4 | 17.0 | 46.3 | 3.2 | error |
| S344V0765 | Humerus | -21.3 | 13.7 | 15.0 | 41.9 | 3.3 | error | -21.5 | 13.2 | 15.4 | 41.7 | 3.2 | error |
| S102V0151 | Humerus | -20.0 | 14.4 | 16.2 | 44.6 | 3.2 | 10.4 | -19.8 | 15.3 | 16.3 | 45.5 | 3.2 | 5.4 |
| S138V0499 | Tibia | -21.0 | 14.6 | 16.6 | 46.7 | 3.3 | error | -20.7 | 15.3 | 15.2 | 43.1 | 3.3 | error |
| S139V0215 | Humerus | -20.4 | 15.2 | 13.4 | 38.6 | 3.4 | 11.6 | -20.2 | 15.6 | 17.5 | 47.5 | 3.2 | 6.2 |
| S376V0900 | Humerus | -21.1 | 13.9 | 15.3 | 42.0 | 3.2 | 21.0 | -21.0 | 13.8 | 15.5 | 42.7 | 3.2 | 24.0 |
| S230V0302 | Humerus | -20.2 | 13.9 | 16.6 | 45.8 | 3.2 | 5.8 | -20.2 | 14.3 | 17.0 | 46.6 | 3.2 | 5.5 |
| S418V0906 | Humerus | -19.0 | 13.9 | 16.7 | 46.1 | 3.2 | 10.9 | -19.7 | 13.9 | 16.3 | 47.4 | 3.4 | 11.7 |
| S274V0418 | Rib | -- | -- | -- | -- | -- | -- | -20.3 | 14.7 | 16.3 | 45.2 | 3.2 | 11.3 |
| S232V0307 | Radius | -20.8 | 14.3 | 16.3 | 45.1 | 3.2 | 2.9 | -20.8 | 14.4 | 16.7 | 46.1 | 3.2 | 9.9 |
| S72V0001 | Humerus | -19.4 | 11.8 | 14.3 | 39.0 | 3.2 | 12.8 | -19.9 | 12.2 | 15.3 | 41.8 | 3.2 | 20.3 |
| S320V0662 | Humerus | -20.7 | 13.5 | 15.5 | 42.5 | 3.2 | 11.8 | -20.5 | 14.1 | 17.1 | 47.1 | 3.2 | 15.7 |
| S191V0374 | Humerus | -20.5 | 13.3 | 16.5 | 45.9 | 3.2 | 5.9 | -20.4 | 13.3 | 16.6 | 45.4 | 3.2 | 5.7 |
| S295V0485 | Humerus | -20.8 | 14.7 | 15.0 | 41.4 | 3.2 | 22.7 | -20.5 | 14.9 | 15.0 | 41.1 | 3.2 | 24.1 |
| S296V0486 | Humerus | -20.8 | 15.0 | 14.6 | 41.1 | 3.3 | 21.0 | -20.6 | 14.6 | 14.8 | 41.0 | 3.2 | 23.9 |
| S164V0364 | Humerus | -19.4 | 14.4 | 16.5 | 47.1 | 3.3 | 8.1 | -19.7 | 14.5 | 16.2 | 46.4 | 3.3 | 11.0 |
| S406V0884 | Humerus | -20.5 | 14.4 | 14.8 | 40.4 | 3.2 | 17.4 | -20.4 | 14.2 | 14.9 | 41.4 | 3.2 | 22.1 |
| S245V0390 | Humerus | -20.2 | 14.5 | 16.4 | 45.6 | 3.2 | error | -20.2 | 14.5 | 16.7 | 45.6 | 3.2 | 11.1 |
| S323V0650 | Humerus | -20.3 | 13.1 | 17.0 | 46.3 | 3.2 | 9.9 | -20.7 | 13.6 | 17.3 | 46.5 | 3.1 | 12.6 |
| S227V0297 | Humerus | -20.4 | 13.9 | 14.9 | 41.5 | 3.2 | 18.9 | -20.5 | 13.7 | 14.9 | 41.1 | 3.2 | 23.5 |
| S373V0798 | Humerus | -20.6 | 14.1 | 15.6 | 43.1 | 3.2 | 19.8 | -21.5 | 14.1 | 14.5 | 40.3 | 3.2 | 24.3 |
| S315V0656 | Humerus | -20.7 | 12.8 | 14.8 | 40.7 | 3.2 | n/a (vial broke) | -20.7 | 13.1 | 17.0 | 46.0 | 3.2 | 12.1 |
| S82V0084 | Humerus | -- | -- | -- | -- | -- | -- | -21.1 | 14.3 | 15.4 | 42.2 | 3.2 | 24.3 |
| S50V0042 | Humerus | -19.3 | 14.2 | 14.9 | 41.5 | 3.2 | 16.7 | -20.6 | 14.1 | 15.3 | 43.1 | 3.3 | 21.0 |
| S0V1524 | Humerus | -20.3 | 12.7 | 16.8 | 45.8 | 3.2 | 13.5 | -20.5 | 13.5 | 16.4 | 45.1 | 3.2 | 15.4 |
| S335V0711 | Humerus | -20.1 | 15.0 | 16.8 | 46.3 | 3.2 | 12.3 | -20.1 | 16.1 | 16.9 | 46.4 | 3.2 | 9.8 |
| S400V0859 | Humerus | -20.5 | 14.5 | 15.5 | 42.7 | 3.2 | 15.7 | -20.4 | 14.7 | 16.2 | 42.4 | 3.1 | 16.1 |
| S0V0358 | Humerus | -20.8 | 14.2 | 14.7 | 42.0 | 3.3 | error | -20.2 | 15.8 | 16.0 | 44.6 | 3.2 | error |
| S130V0173 | Humerus | -20.2 | 15.2 | 16.1 | 44.6 | 3.2 | error | -20.1 | 15.3 | 17.1 | 47.1 | 3.2 | 9.5 |
| S187V0267 | Humerus | -21.1 | 13.6 | 16.3 | 45.6 | 3.3 | error | -21.1 | 13.3 | 16.3 | 44.9 | 3.2 | error |
| S273V0619 | Femur | -19.9 | 14.0 | 17.2 | 47.0 | 3.2 | error | -20.0 | 14.4 | 16.4 | 45.2 | 3.2 | 4.9 |
| S287V0450 | Humerus | -20.9 | 14.3 | 16.6 | 45.6 | 3.2 | 7.0 | -21.1 | 14.4 | 16.6 | 45.6 | 3.2 | 12.4 |
| S99V0139 | Humerus | -16.0 | 13.0 | 16.9 | 47.5 | 3.3 | 23.2 | -18.4 | 14.2 | 17.0 | 46.9 | 3.2 | 22.0 |
| S133V0299 | Humerus | -19.3 | 14.1 | 16.8 | 47.2 | 3.3 | 5.9 | -20.0 | 14.5 | 14.9 | 39.0 | 3.1 | 15.7 |
| S352V0747 | Humerus | -19.4 | 14.8 | 16.8 | 47.4 | 3.3 | 9.3 | -20.2 | 14.3 | 17.1 | 46.8 | 3.2 | 13.6 |
| S493V1069 | Humerus | -20.9 | 13.6 | 16.0 | 45.7 | 3.3 | error | -21.3 | 14.3 | 15.9 | 44.5 | 3.3 | error |
| S214V0227 | Tibia | -19.1 | 14.4 | 16.4 | 46.2 | 3.3 | 9.0 | -19.9 | 14.0 | 17.2 | 47.1 | 3.2 | 16.3 |
| S330V0706 | Parietal Fragment | -- | -- | -- | -- | -- | -- | -19.6 | 14.6 | 16.3 | 45.2 | 3.2 | 14.6 |
| S314V0655 | Humerus | -19.8 | 11.9 | 15.5 | 42.5 | 3.2 | 12.4 | -19.8 | 12.6 | 17.1 | 46.7 | 3.2 | 13.4 |
| S152V0244 | Humerus | -20.0 | 15.4 | 16.0 | 43.7 | 3.2 | 19.9 | -20.0 | 15.7 | 15.9 | 43.4 | 3.2 | 22.2 |
| S103V0153 | Femur | -- | -- | -- | -- | -- | -- | -18.5 | 12.8 | 16.4 | 47.4 | 3.4 | 8.7 |
| S37V0021 | Humerus | -19.7 | 13.8 | 16.0 | 44.8 | 3.3 | 19.6 | -20.5 | 14.5 | 16.5 | 45.3 | 3.2 | 25.2 |
| S421V0940 | Rib | -- | -- | -- | -- | -- | -- | -19.7 | 15.4 | 16.7 | 46.6 | 3.3 | 18.0 |
| S122V0161 | Tibia | -18.9 | 14.4 | 16.5 | 47.3 | 3.3 | 10.3 | -18.3 | 13.8 | 17.4 | 47.7 | 3.2 | 20.1 |
| S190V0310 | Humerus | -18.8 | 14.8 | 16.7 | 45.9 | 3.2 | 7.8 | -18.8 | 14.4 | 16.9 | 46.3 | 3.2 | 12.7 |
| S86V0106 | Tibia | -19.1 | 16.6 | 16.1 | 46.1 | 3.3 | 12.1 | -19.2 | 15.3 | 15.3 | 42.4 | 3.2 | 16.6 |
| S80V0049 | Clavicle | -19.3 | 12.8 | 17.1 | 47.8 | 3.3 | 7.2 | -19.5 | 13.5 | 17.3 | 47.2 | 3.2 | 10.1 |
| S91V0110 | Femur | -- | -- | -- | -- | -- | -- | -18.7 | 12.9 | 16.5 | 46.4 | 3.3 | 12.3 |
| S241V0359 | Humerus | -21.5 | 13.2 | 14.8 | 40.8 | 3.2 | 22.0 | -21.1 | 13.8 | 14.9 | 40.7 | 3.2 | 24.2 |
| S231V0305 | Rib | -- | -- | -- | -- | -- | -- | -20.8 | 14.1 | 17.2 | 46.9 | 3.2 | 20.1 |
| S58V0092 | Humerus | -18.5 | 13.1 | 16.0 | 45.2 | 3.3 | 12.6 | -18.0 | 13.8 | 16.9 | 46.8 | 3.2 | 16.0 |
| S106V0142 | Tibia | -20.5 | 13.2 | 15.2 | 44.4 | 3.4 | 17.4 | -20.3 | 12.9 | 15.5 | 42.3 | 3.2 | 19.0 |
| S38V0026 | Humerus | -21.4 | 12.8 | 14.3 | 37.9 | 3.2 | 21.5 | -21.4 | 13.3 | 12.3 | 36.0 | 3.4 | 25.5 |
| S158V0230 | Humerus | -21.1 | 12.9 | 16.3 | 46.1 | 3.3 | 21.0 | -20.5 | 12.7 | 16.5 | 46.9 | 3.3 | 20.5 |
| S24V0086 | Femur | -19.5 | 13.5 | 16.6 | 45.7 | 3.2 | 18.7 | -19.3 | 13.5 | 16.3 | 45.0 | 3.2 | 14.3 |
| S44V0020 | Humerus | -20.4 | 13.1 | 16.2 | 44.8 | 3.2 | 8.0 | -20.6 | 13.3 | 16.3 | 46.3 | 3.3 | 11.1 |
| S35V0031 | Humerus | -20.7 | 13.9 | 17.7 | 48.5 | 3.2 | n/a (vial broke) | -20.7 | 13.4 | 16.4 | 45.8 | 3.3 | 10.8 |
| S127V0204 | Humerus | -20.1 | 13.7 | 15.5 | 42.9 | 3.2 | 18.0 | -20.0 | 13.5 | 18.0 | 48.7 | 3.2 | 16.3 |
| S318V0643 | Humerus | -20.8 | 12.6 | 16.6 | 46.4 | 3.3 | 8.6 | -21.8 | 13.4 | 14.7 | 41.5 | 3.3 | error |
| S165V0242 | Humerus | -21.3 | 14.3 | 16.2 | 44.9 | 3.2 | 12.8 | -21.0 | 14.7 | 16.1 | 44.7 | 3.2 | 11.2 |
| S46V0023 | Radius | -21.0 | 13.8 | 14.9 | 41.8 | 3.3 | 13.9 | -20.9 | 15.0 | 16.5 | 45.2 | 3.2 | 17.3 |
| S284V0466 | Humerus | -19.5 | 14.2 | 17.6 | 48.2 | 3.2 | 12.0 | -20.3 | 13.2 | 17.4 | 47.4 | 3.2 | 19.1 |
| S328V0701 | Humerus | -21.1 | 14.5 | 15.1 | 41.4 | 3.2 | 13.7 | -20.6 | 14.9 | 15.9 | 42.5 | 3.1 | 20.7 |
| S351V0756 | Humerus | -20.5 | 12.5 | 16.9 | 46.0 | 3.2 | 12.0 | -20.5 | 12.9 | 17.4 | 46.4 | 3.1 | 16.3 |
| S343V0732 | Humerus | -21.2 | 13.2 | 14.9 | 41.4 | 3.2 | 24.0 | -- | -- | -- | -- | -- | -- |
| S62V0071 | Radius | -20.6 | 14.7 | 14.9 | 42.1 | 3.3 | 18.2 | -21.1 | 15.5 | 17.2 | 47.2 | 3.2 | 23.9 |
| S177V0413 | Humerus | -19.7 | 14.4 | 16.4 | 45.5 | 3.2 | 13.7 | -19.7 | 14.2 | 17.4 | 47.6 | 3.2 | 16.7 |
| S252V0443 | Femur | -20.2 | 13.7 | 17.3 | 47.6 | 3.2 | 16.9 | -19.9 | 14.0 | 17.9 | 48.8 | 3.2 | 19.0 |
| S89V0091 | Humerus | -21.6 | 14.9 | 18.0 | 50.9 | 3.3 | error | -21.5 | 15.9 | 16.6 | 47.8 | 3.4 | 11.5 |
| S145V0238 | Tibia | -19.3 | 12.2 | 16.8 | 46.3 | 3.2 | 7.2 | -20.1 | 12.7 | 17.2 | 47.3 | 3.2 | 15.6 |
| S255V0398 | Humerus | -21.1 | 11.9 | 17.4 | 48.0 | 3.2 | 13.1 | -20.6 | 13.0 | 17.1 | 46.7 | 3.2 | 18.3 |
| S316V0641 | Humerus | -20.7 | 12.6 | 16.0 | 43.6 | 3.2 | 15.5 | -20.7 | 12.8 | 17.2 | 46.2 | 3.1 | 20.7 |
| S189V0332 | Radius | -17.7 | 13.9 | 16.1 | 44.1 | 3.2 | 5.6 | -20.3 | 13.9 | 17.0 | 46.3 | 3.2 | error |
| S32V0082 | Humerus | -21.4 | 13.6 | 15.6 | 42.7 | 3.2 | 21.4 | -21.2 | 12.9 | 15.2 | 43.0 | 3.3 | 12.3 |
| S141V0223 | Humerus | -20.7 | 14.8 | 15.1 | 41.8 | 3.2 | 10.1 | -20.8 | 14.6 | 17.2 | 47.1 | 3.2 | 16.4 |
| S293V0495 | Humerus | -20.6 | 13.8 | 15.6 | 42.8 | 3.2 | 25.5 | -20.1 | 14.9 | 16.0 | 47.9 | 3.5 | 22.5 |
| S326V0708 | Humerus | -20.9 | 12.3 | 15.9 | 44.1 | 3.2 | 18.1 | -21.0 | 13.0 | 16.3 | 45.1 | 3.2 | 19.6 |
| S463V0988 | Humerus | -21.1 | 13.5 | 15.6 | 44.5 | 3.3 | 10.1 | -20.5 | 13.3 | 17.4 | 46.8 | 3.1 | 19.2 |
| S109V0175 | Femur | -20.6 | 13.5 | 17.7 | 50.3 | 3.3 | 20.1 | -20.5 | 12.9 | 17.3 | 46.9 | 3.2 | 19.2 |
| S104V0136 | Humerus | -20.6 | 13.1 | 15.8 | 42.4 | 3.1 | 14.3 | -21.0 | 13.1 | 17.5 | 49.2 | 3.3 | 18.2 |
| S105V0170 | Tibia | -20.6 | 13.4 | 15.3 | 41.1 | 3.1 | 13.5 | -20.7 | 13.1 | 17.3 | 48.3 | 3.3 | 17.4 |
| S215V0250 | Radius | -20.9 | 13.8 | 16.9 | 47.0 | 3.2 | 16.9 | -20.6 | 14.1 | 16.9 | 46.3 | 3.2 | 15.3 |
| S181V0407 | Humerus | -21.1 | 12.5 | 17.7 | 49.8 | 3.3 | 17.0 | -21.4 | 12.9 | 15.6 | 44.6 | 3.3 | 3.0 |
| S353V0739 | Humerus | -20.5 | 13.3 | 16.8 | 48.8 | 3.4 | 16.2 | -20.5 | 14.5 | 16.0 | 45.0 | 3.3 | 9.3 |
| S140V0207 | Humerus | -20.1 | 14.0 | 16.8 | 47.0 | 3.3 | 11.1 | -20.5 | 13.9 | 15.9 | 45.2 | 3.3 | 1.7 |
| S336V0709 | Humerus | -20.5 | 13.6 | 15.6 | 49.2 | 3.2 | 22.4 | -20.9 | 13.7 | 12.5 | 37.0 | 3.5 | 13.6 |

| ID Number | Bone Analysed | δ^13^C (‰) | δ^15^N (‰) | %N by weight | %C by weight | C/N ratio | collagen yield |
| --- | --- | --- | --- | --- | --- | --- | --- |
| Juveniles (7-12 years) | | | | | | | |
| S362V0770 | Rib | -20.3 | 13.6 | 17.5 | 48.4 | 3.2 | 20.9 |
| S389V0857 | Rib | -20.5 | 13.6 | 15.1 | 40.9 | 3.2 | 19.8 |
| S18V0102 | Rib | -20.7 | 12.8 | 17.7 | 47.1 | 3.2 | 16.5 |
| S273V0611 | Rib | -20.6 | 12.7 | 17.0 | 46.9 | 3.2 | 16.3 |
| S365V0773 | Rib | -21.0 | 13.1 | 15.1 | 40.5 | 3.1 | 17.4 |
| S384V0839 | Rib | -20.9 | 12.9 | 17.0 | 46.8 | 3.2 | 22.9 |
| S396V0877 | Humerus | -20.9 | 14.8 | 14.0 | 38.0 | 3.2 | 10.7 |
| S248V0393 | Rib | -20.9 | 14.3 | 17.4 | 47.3 | 3.2 | 18.7 |
| S188V0415 | Rib | -21.3 | 13.2 | 16.2 | 45.6 | 3.3 | 8.2 |
| S471V1020 | Rib | -21.4 | 13.0 | 16.5 | 46.7 | 3.3 | 21.0 |
| S480V1042 | Rib | -20.8 | 13.6 | 17.2 | 47.4 | 3.2 | 18.8 |
| S503V1099 | Rib | -20.5 | 13.5 | 17.0 | 47.0 | 3.2 | 21.6 |
| S515V1111 | Rib | -20.4 | 13.8 | 17.2 | 47.9 | 3.2 | 21.5 |
| S334V0716 | Humerus | -20.5 | 13.6 | 14.9 | 41.0 | 3.2 | 24.2 |
| S286V0469 | Humerus | -21.0 | 13.2 | 15.0 | 49.2 | 3.3 | error |
| S269V1506 | Rib | -20.8 | 13.0 | 17.2 | 46.7 | 3.2 | 17.0 |
| S450V1154 | Rib | -20.7 | 13.1 | 16.8 | 46.8 | 3.3 | 10.8 |
| S128V0180 | Rib | -20.5 | 13.1 | 17.1 | 49.2 | 3.4 | 19.6 |
| S367V0803 | Rib | -21.0 | 12.7 | 15.5 | 41.2 | 3.1 | 18.8 |
| S549V1181 | Rib | -20.7 | 14.0 | 16.5 | 46.3 | 3.3 | 12.0 |
| S269V1065 | Humerus | -20.6 | 12.4 | 17.4 | 47.4 | 3.2 | 18.5 |
| S256V0462 | Femur | -21.1 | 12.7 | 17.3 | 47.4 | 3.2 | 19.8 |
| S44V0027 | Rib | -20.2 | 13.3 | 17.4 | 50.5 | 3.4 | 22.9 |
| S282V0417 | Rib | -20.5 | 14.2 | 17.2 | 47.2 | 3.2 | 20.8 |
| S167V0270 | Rib | -20.1 | 14.3 | 16.7 | 46.5 | 3.3 | 20.3 |
| Adolescents (13-17 years) | | | | | | | |
| S196V0437 | Rib | -20.8 | 12.3 | 17.3 | 47.5 | 3.2 | 20.4 |
| S522V1127 | Rib | -21.3 | 13.3 | 16.8 | 46.9 | 3.3 | 23.0 |
| S479V1019 | Rib | -21.2 | 12.2 | 17.0 | 46.9 | 3.2 | 17.0 |
| S507V1093 | Rib | -20.9 | 13.5 | 16.2 | 45.1 | 3.3 | 9.8 |
| S275V0526 | Rib | -20.4 | 13.6 | 17.5 | 47.5 | 3.2 | 20.7 |
| S465V1001 | Rib | -21.1 | 13.2 | 16.2 | 42.2 | 3.0 | 26.2 |
| S123V0182 | Rib | -20.5 | 12.1 | 17.4 | 47.2 | 3.2 | 18.6 |
| S462V0987 | Rib | -20.8 | 13.7 | 17.1 | 47.3 | 3.2 | 19.7 |
| S446V0944 | Rib | -21.2 | 13.6 | 16.3 | 46.3 | 3.3 | 8.1 |
| S460V0971 | Rib | -20.5 | 13.0 | 16.0 | 42.1 | 3.1 | 19.2 |
| S452V0985 | Rib | -21.1 | 13.2 | 17.1 | 47.3 | 3.2 | 23.1 |
| S229V0324 | Rib | -20.4 | 13.7 | 16.9 | 47.1 | 3.3 | 21.4 |
| S278V0474 | Rib | -19.7 | 10.7 | 15.1 | 41.6 | 3.2 | 20.0 |
| Young Adult (18-34 years) Females | | | | | | | |
| S47V0045 | Rib | -20.7 | 13.0 | 14.5 | 40.3 | 3.2 | 23.3 |
| S311V0956 | Rib | -20.8 | 13.4 | 16.1 | 43.8 | 3.2 | 20.2 |
| S344V0730 | Rib | -20.5 | 13.6 | 14.9 | 41.0 | 3.2 | 21.0 |
| S307V0591 | Manual Phalanx | -20.2 | 14.4 | 14.7 | 41.1 | 3.3 | 25.2 |
| S388V0952 | Rib | -20.5 | 13.5 | 15.9 | 41.6 | 3.1 | 24.1 |
| S350V0844 | Rib | -20.1 | 11.1 | 15.0 | 41.4 | 3.2 | 25.1 |
| S149V0280 | Rib | -21.2 | 13.1 | 16.9 | 46.3 | 3.2 | 16.4 |
| S385V0874 | Manual Phalanx | -21.0 | 13.8 | 16.2 | 45.3 | 3.3 | 22.5 |
| S372V0808 | Manual Phalanx | -20.7 | 13.0 | 16.1 | 41.9 | 3.0 | 24.2 |
| S370V0806 | Rib | -20.3 | 13.9 | 17.7 | 52.2 | 3.4 | 21.9 |
| S192V0636 | Manual Phalanx | -20.6 | 13.8 | 16.5 | 46.3 | 3.3 | 18.4 |
| S88V0094 | Rib | -21.1 | 14.2 | 15.2 | 41.5 | 3.2 | 25.6 |
| S327V0758 | Rib | -20.8 | 13.7 | 14.7 | 40.9 | 3.2 | 24.8 |
| S60V0037 | Rib | -21.1 | 13.7 | 17.1 | 47.4 | 3.2 | 14.5 |
| S151V0666 | Rib | -21.0 | 13.8 | 14.6 | 40.2 | 3.2 | 18.6 |
| S382V0818 | Rib | -20.3 | 13.7 | 17.4 | 47.1 | 3.2 | 20.1 |
| S345V0757 | Rib | -20.9 | 13.5 | 14.8 | 41.2 | 3.2 | 17.3 |
| S461V0990 | Rib | -21.0 | 12.7 | 16.1 | 42.1 | 3.1 | 21.9 |
| S160V0613 | Rib | -21.1 | 13.6 | 14.8 | 40.3 | 3.2 | 25.0 |
| S481V1046 | Rib | -20.5 | 14.7 | 16.7 | 46.7 | 3.3 | 12.1 |
| S487V1096 | Rib | -20.6 | 14.4 | 16.2 | 44.9 | 3.2 | 17.9 |
| S422V0962 | Rib | -20.3 | 13.6 | 14.3 | 39.3 | 3.2 | 19.5 |
| S198V0601 | Rib | -20.9 | 13.6 | 17.2 | 47.4 | 3.2 | 13.9 |
| S430V0965 | Rib | -20.9 | 13.3 | 17.3 | 47.6 | 3.2 | 17.4 |
| S369V0886 | Rib | -20.8 | 13.5 | 16.6 | 46.4 | 3.3 | 13.7 |
| S476V1054 | Rib | -20.7 | 14.5 | 17.1 | 47.2 | 3.2 | 20.7 |
| S453V0973 | Rib | -21.0 | 13.5 | 17.0 | 47.1 | 3.2 | 20.4 |
| S338V0721 | Rib | -21.0 | 14.7 | 14.5 | 41.0 | 3.3 | 18.1 |
| S107V0148 | Rib | -20.3 | 13.6 | 14.6 | 40.1 | 3.2 | 21.8 |
| S183V0311 | Rib | -20.1 | 13.6 | 17.1 | 47.3 | 3.2 | 16.6 |
| S527V1053 | Rib | -20.7 | 14.7 | 15.7 | 42.9 | 3.2 | 22.2 |
| Middle Adult Females (35-49 years) | | | | | | | |
| S84V0113 | Rib | -21.0 | 13.9 | 16.0 | 42.4 | 3.1 | 16.9 |
| S278V0584 | Rib | -21.1 | 14.3 | 15.6 | 42.4 | 3.2 | 23.7 |
| S485V1034 | Rib | -20.4 | 14.3 | 15.5 | 42.8 | 3.2 | 23.6 |
| S213V0220 | Rib | -20.8 | 13.5 | 16.8 | 46.5 | 3.2 | 20.3 |
| S529V1138 | Rib | -20.8 | 12.9 | 16.2 | 42.1 | 3.0 | 21.2 |
| S524V1120 | Rib | -20.7 | 14.5 | 16.6 | 46.3 | 3.2 | 19.7 |
| S101V0131 | Rib | -21.0 | 13.4 | 13.0 | 35.6 | 3.2 | 20.3 |
| S413V0896 | Rib | -20.4 | 13.3 | 16.1 | 42.3 | 3.1 | 17.8 |
| S387V0914 | Fibula | -20.5 | 13.9 | 15.7 | 41.5 | 3.1 | 19.8 |
| S466V1010 | Rib | -20.8 | 14.5 | 15.9 | 41.7 | 3.1 | 22.5 |
| S303V0520 | Rib | -20.4 | 13.9 | 16.8 | 46.8 | 3.2 | 22.0 |
| S174V0408 | Rib | -21.1 | 13.5 | 14.8 | 40.9 | 3.2 | 21.0 |
| S359V0760 | Rib | -20.8 | 12.5 | 16.3 | 42.5 | 3.0 | 22.2 |
| S45V0055 | Rib | -20.9 | 14.0 | 14.9 | 40.7 | 3.2 | 25.8 |
| S137V0491 | Rib | -20.6 | 14.1 | 16.1 | 46.8 | 3.4 | 14.6 |
| S545V1178 | Rib | -20.4 | 13.7 | 17.5 | 48.3 | 3.2 | 16.4 |
| Old Adult (50+ years) Females | | | | | | | |
| S157V0470 | Rib | -20.4 | 14.5 | 14.7 | 40.8 | 3.2 | 24.7 |
| S170V0660 | Rib | -19.9 | 14.1 | 15.1 | 41.2 | 3.2 | 25.1 |
| S195V0588 | Manual Phalanx | -20.5 | 13.7 | 16.9 | 47.0 | 3.3 | 16.2 |
| S155V1509 | Rib | -21.1 | 13.9 | 17.6 | 48.4 | 3.2 | 26.9 |
| S426V0968 | Rib | -20.8 | 13.3 | 15.8 | 43.7 | 3.2 | 20.5 |
| S383V0880 | Rib | -20.8 | 13.9 | 17.2 | 48.0 | 3.2 | 16.3 |
| S53V0290 | Rib | -20.1 | 15.5 | 17.0 | 47.4 | 3.2 | 18.2 |
| S457V0960 | Rib | -20.7 | 13.7 | 16.8 | 46.6 | 3.2 | 20.0 |
| S394V0869 | Rib | -20.5 | 14.3 | 17.5 | 48.7 | 3.2 | 21.0 |
| S77V0098 | Manual Phalanx | -21.0 | 13.9 | 17.3 | 48.3 | 3.2 | 19.7 |
| S309V0616 | Rib | -20.7 | 14.5 | 15.1 | 41.2 | 3.2 | 18.5 |
| S346V0733 | Rib | -19.3 | 14.7 | 14.9 | 41.5 | 3.2 | 24.2 |
| S386V0848 | Manual Phalanx | -20.9 | 13.4 | 15.5 | 41.3 | 3.1 | 21.4 |
| S243V0381 | Rib | -21.0 | 13.8 | 16.6 | 45.2 | 3.2 | 15.0 |
| S436V0911 | Rib | -20.5 | 14.4 | 16.0 | 44.0 | 3.2 | 16.5 |
| S339V0728 | Rib | -20.9 | 13.5 | 14.9 | 40.2 | 3.1 | 20.3 |
| S428V0945 | Rib | -20.6 | 14.5 | 15.9 | 41.9 | 3.1 | 21.7 |
| S360V0762 | Rib | -20.4 | 15.0 | 16.2 | 42.4 | 3.0 | 22.2 |
| S294V0487 | Rib | -20.8 | 13.3 | 17.3 | 47.8 | 3.2 | 20.6 |
| S126V0184 | Rib | -21.6 | 13.6 | 17.2 | 47.5 | 3.2 | 14.2 |
| S486V1088 | Manual Phalanx | -20.8 | 14.0 | 16.5 | 46.4 | 3.3 | 18.7 |
| S319V0669 | Rib | -20.0 | 14.0 | 15.5 | 42.6 | 3.2 | 23.7 |
| S390V0831 | Rib | -19.2 | 14.3 | 16.4 | 45.3 | 3.2 | 20.5 |
| S302V0509 | Rib | -20.5 | 14.7 | 15.5 | 42.4 | 3.2 | 23.3 |
| S413V0895 | Rib | -20.9 | 13.7 | 16.3 | 42.8 | 3.1 | 24.1 |
| S358V0763 | Rib | -20.5 | 14.5 | 16.1 | 42.2 | 3.1 | 16.7 |
| S331V0735 | Rib | -20.5 | 14.0 | 14.6 | 40.8 | 3.3 | 22.5 |
| S530V1159 | Rib | -21.1 | 13.4 | 17.2 | 47.6 | 3.2 | 19.8 |
| S159V0200 | Fibula | -20.4 | 13.7 | 17.3 | 47.2 | 3.2 | 21.1 |
| S97V0156 | Rib | -20.5 | 14.4 | 14.4 | 40.3 | 3.3 | 25.6 |
| S56V0061 | Pedal Phalanx | -20.1 | 15.3 | 17.2 | 47.7 | 3.2 | 19.4 |
| S356V0864 | Rib | -20.7 | 13.7 | 16.9 | 48.4 | 3.3 | 20.3 |
| S501V1097 | Rib | -20.9 | 14.1 | 16.7 | 46.3 | 3.2 | 20.9 |
| Young Adult (18-34 years) Males | | | | | | | |
| S290V0472 | Rib | -20.7 | 13.9 | 15.2 | 41.5 | 3.2 | 22.7 |
| S246V0396 | Rib | -20.9 | 13.0 | 17.1 | 46.9 | 3.2 | 20.6 |
| S340V0724 | Rib | -20.8 | 12.8 | 16.1 | 44.7 | 3.2 | 19.1 |
| S540V1172 | Rib | -21.2 | 13.0 | 16.9 | 47.1 | 3.3 | 21.1 |
| S454V0963 | Rib | -20.2 | 13.3 | 16.0 | 42.2 | 3.1 | 18.6 |
| S239V0369 | Rib | -20.9 | 13.6 | 15.5 | 42.1 | 3.2 | 27.0 |
| S236V0335 | Rib | -20.8 | 13.8 | 17.0 | 47.2 | 3.2 | 19.4 |
| S40V0064 | Rib | -20.6 | 12.9 | 15.2 | 41.7 | 3.2 | 23.2 |
| S180V0433 | Rib | -21.0 | 14.7 | 14.9 | 40.8 | 3.2 | 24.2 |
| S502V1062 | Rib | -21.5 | 13.6 | 15.5 | 45.2 | 3.4 | 5.5 |
| S404V1139 | Rib | -21.0 | 14.3 | 15.3 | 43.0 | 3.3 | 20.6 |
| S492V1039 | Rib | -20.6 | 14.2 | 15.6 | 42.6 | 3.2 | 22.0 |
| S249V0394 | Rib | -21.0 | 13.1 | 15.0 | 41.0 | 3.2 | 24.7 |
| S427V0938 | Rib | -20.6 | 13.6 | 16.0 | 42.4 | 3.1 | 19.1 |
| S544V1227 | Rib | -20.4 | 12.9 | 15.9 | 40.6 | 3.0 | 23.6 |
| S306V0561 | Rib | -20.9 | 13.0 | 15.2 | 41.6 | 3.2 | 23.1 |
| S251V0624 | Rib | -19.9 | 15.7 | 14.8 | 40.7 | 3.2 | 25.4 |
| Middle Adult (35-49 years) Males | | | | | | | |
| S144V0222 | Rib | -19.8 | 14.0 | 17.1 | 47.2 | 3.2 | 20.3 |
| S240V0362 | Rib | -20.9 | 13.7 | 15.2 | 41.2 | 3.2 | 23.7 |
| S263V0445 | Rib | -20.8 | 13.1 | 15.7 | 43.0 | 3.2 | 22.1 |
| S399V0872 | Rib | -21.0 | 13.2 | 16.0 | 42.6 | 3.1 | 24.9 |
| S411V0904 | Rib | -20.4 | 14.0 | 16.1 | 42.2 | 3.1 | 19.0 |
| S464V1012 | Rib | -20.7 | 14.0 | 17.6 | 48.5 | 3.2 | 18.2 |
| S281V0542 | Rib | -20.5 | 13.0 | 15.5 | 42.2 | 3.2 | 22.3 |
| S173V0198 | Rib | -20.8 | 13.6 | 15.2 | 41.5 | 3.2 | 20.0 |
| S350V0838 | Rib | -20.0 | 14.2 | 14.5 | 40.5 | 3.3 | 7.3 |
| S470V1026 | Rib | -20.2 | 13.0 | 15.4 | 42.5 | 3.2 | 18.2 |
| S472V0994 | Rib | -20.8 | 14.8 | 15.6 | 42.9 | 3.2 | 22.4 |
| S534V1165 | Rib | -20.5 | 13.4 | 15.4 | 40.1 | 3.0 | 24.7 |
| S482V1048 | Rib | -20.5 | 13.5 | 15.6 | 43.0 | 3.2 | 22.7 |
| S325V0676 | Rib | -20.3 | 14.4 | 16.3 | 45.4 | 3.3 | 16.9 |
| S59V0133 | Rib | -20.6 | 13.5 | 18.2 | 50.0 | 3.2 | 20.1 |
| S473V1003 | Rib | -20.4 | 14.5 | 17.0 | 47.1 | 3.2 | 20.2 |
| S435V0929 | Rib | -21.1 | 12.5 | 17.4 | 48.4 | 3.2 | 20.7 |
| S313V0926 | Rib | -21.0 | 14.2 | 17.5 | 47.9 | 3.2 | 20.3 |
| Old Adult (50+ years) Males | | | | | | | |
| S496V1043 | Manual Phalnax | -20.9 | 13.2 | 16.5 | 44.8 | 3.2 | 17.8 |
| S254V0357 | Rib | -21.1 | 13.1 | 15.4 | 42.0 | 3.2 | 23.7 |
| S278V0522 | Rib | -20.3 | 10.1 | 15.4 | 42.6 | 3.2 | 18.9 |
| S469V1016 | Rib | -21.0 | 13.9 | 15.5 | 43.1 | 3.2 | 22.2 |
| S324V0671 | Rib | -20.9 | 14.0 | 16.1 | 42.5 | 3.1 | 23.9 |
| S162V0316 | Rib | -20.8 | 13.9 | 15.1 | 41.1 | 3.2 | 25.3 |
| S289V0477 | Rib | -21.3 | 14.4 | 17.1 | 47.0 | 3.2 | 21.0 |
| S347V0741 | Rib | -20.9 | 13.5 | 16.3 | 42.3 | 3.0 | 27.0 |
| S153V0435 | Rib | -21.1 | 14.1 | 15.2 | 41.4 | 3.2 | 22.5 |
| S194V0440 | Rib | -21.2 | 13.6 | 17.2 | 47.2 | 3.2 | 20.3 |
| S92V0124 | Rib | -21.1 | 13.0 | 14.7 | 40.5 | 3.2 | 22.9 |
| S158V0427 | Rib | -20.1 | 14.2 | 15.1 | 41.2 | 3.2 | 25.3 |
| S477V1030 | Rib | -20.2 | 14.6 | 17.1 | 47.2 | 3.2 | 19.6 |
| S363V0766 | Rib | -20.8 | 14.0 | 16.1 | 42.4 | 3.1 | 22.9 |
| S34V0074 | Rib | -20.7 | 14.3 | 14.8 | 40.8 | 3.2 | 22.9 |
| S93V0126 | Rib | -20.6 | 14.9 | 16.9 | 47.0 | 3.3 | 18.0 |
| S381V0824 | Manual Phalanx | -19.3 | 16.3 | 16.7 | 47.1 | 3.3 | 20.6 |
| S337V0714 | Rib | -20.5 | 14.8 | 14.8 | 40.9 | 3.2 | 19.0 |
| S521V1150 | Rib | -20.3 | 12.8 | 15.6 | 42.4 | 3.2 | n/a (vial broke) |
| S285V0452 | Rib | -20.6 | 14.0 | 14.8 | 41.0 | 3.2 | 19.8 |
| S520V1118 | Rib | -20.1 | 15.7 | 16.9 | 47.1 | 3.3 | 15.2 |
| S250V0402 | Rib | -21.0 | 14.7 | 17.2 | 47.3 | 3.2 | 18.5 |
| S375V0815 | Rib | -20.7 | 14.5 | 16.0 | 41.7 | 3.0 | 24.1 |
| S51V0059 | Rib | -21.1 | 13.7 | 16.1 | 43.8 | 3.2 | 16.4 |
| S342V0737 | Rib | -20.6 | 13.5 | 16.1 | 42.0 | 3.0 | 20.1 |
| S100V0159 | Rib | -20.7 | 14.4 | 14.8 | 40.9 | 3.2 | 24.5 |
| S357V0745 | Rib | -20.7 | 14.2 | 15.0 | 42.3 | 3.3 | 22.4 |
| S253V0466 | Rib | -20.7 | 13.4 | 15.1 | 41.0 | 3.2 | 16.3 |
| S228V0343 | Manual Phalanx | -20.6 | 13.7 | 14.7 | 40.5 | 3.2 | 22.8 |
| S261V0422 | Manual Phalanx | -20.5 | 14.3 | 17.0 | 47.0 | 3.2 | 19.9 |
| S374V0861 | Rib | -20.5 | 14.2 | 16.1 | 42.2 | 3.1 | 18.4 |
| S317V0649 | Rib | -20.5 | 13.7 | 17.0 | 46.9 | 3.2 | 20.8 |
| S155V0196 | Rib | -20.6 | 15.1 | 17.0 | 47.4 | 3.2 | 18.6 |
| S466V0996 | Rib | -21.2 | 13.9 | 15.9 | 42.0 | 3.1 | 24.0 |
| S322V0651 | Rib | -21.2 | 13.6 | 16.0 | 42.1 | 3.1 | 23.0 |
| S200V0429 | Manual Phalanx | -20.7 | 14.6 | 16.6 | 46.6 | 3.3 | 13.2 |
| S349V0752 | Rib | -21.1 | 13.7 | 15.8 | 42.0 | 3.1 | 20.8 |

^1^error results from an undetected malfunctioning scale during initial scintillation vial weighing. n/a (vial broke) refers to a scintillation vial that broke in the freeze-dryer preventing accurate calculation of collagen yield.
