# Supplementary material for: Isotopic reconstruction of short to absent breastfeeding in a 19th century rural Dutch community
Source: PLoS One. 2022 Apr 13;17(4):e0265821. doi: 10.1371/journal.pone.0265821 (PMC9007374; doi:10.1371/journal.pone.0265821)
Supplement: S3 Table — (DOCX) [file pone.0265821.s003.docx]

Supplement Table 3: Results of statistical tests not specified in main paper

| Comparison | Statistical Test | Sample Size | Result | p-value |
| --- | --- | --- | --- | --- |
| Older Infant+Toddler v. Child v. Juvenile v. Adolescent δ^15^N | ANOVA | Older Infant+Toddler n=14  Child n=22  Juvenile n=25  Adolescent n=13 | F=2.883 | 0.042 |
|  | Tukey post-hoc Older Infant+Toddler v. Adolescent |  | -- | 0.053 |
|  | Tukey post-hoc  Child v. Adolescent |  | -- | 0.109 |
| Older Infant+Toddler v. Child v. Juvenile v. Adolescent δ^13^C | ANOVA | Older Infant+Toddler n=14  Child n=22  Juvenile n=25  Adolescent n=13 | F=0.992 | 0.402 |
| YA v. MA v. OA F δ^15^N | ANOVA | YA F n=31  MA F n=16  OA F n=33 | F=4.438 | 0.015 |
|  | Tukey post-hoc YA v. OA F |  | -- | 0.012 |
|  | Tukey post-hoc YA v. MA F |  | -- | 0.721 |
|  | Tukey post-hoc MA v. OA F |  | -- | 0.221 |
| YA v. MA v. OA F δ^13^C | ANOVA |  | F=1.671 | 0.195 |
| YA v. MA v. OA M δ^15^N | ANOVA | YA M n=17  MA M n=18  OA M n=37 | F=1.328 | 0.271 |
| YA v. MA v. OA M δ^13^C | ANOVA |  | F=1.239 | 0.296 |
| YA+MA v. OA M&F δ^15^N | t-test | YA+MA M&F n=83  OA M&F n=70 | t=-2.984 | 0.003 |
| YA+MA v. OA M&F δ^13^C | t-test |  | t=-0.771 | 0.444 |
| Archivally identified M v. F young infant δ^15^N | Mann-Whitney U | M meta n=5, dia n=6  F meta & dia n=3 | meta U=6.500  dia U=8.00 | meta p=0.764 dia p=0.796 |
| Archivally identified M v. F older infant+toddler δ^15^N | Mann-Whitney U | M meta & dia n=4  F meta & dia n=5 | meta U=4.00  dia U=5.00 | meta p=0.142  dia p=0.219 |
| Archivally identified M v. F child δ^15^N | Mann-Whitney U | M meta & dia n=9  F meta & diap n=5 | meta U=17.50  dia U=13.00 | meta p=0.252 dia p=0.155 |
| Archivally identified M v. F juvenile+adolescent δ^15^N | Mann-Whitney U | M n=5  F n=11 | U=25.00 | p=0.776 |
| Archivally identified M v. F young infant δ^13^C | Mann-Whitney U | M meta n=5, dia n=6  F meta & dia n=3 | meta U=5.00  dia U=8.00 | meta p=0.456  dia p=0.796 |
| Archivally identified M v. F older infant+toddler δ^13^C | Mann-Whitney U | M meta & dia n=4  F meta & dia n=5 | meta U=7.00  dia U=7.00 | meta p=0.461 dia p=0.462 |
| Archivally identified M v. F child δ^13^C | Mann-Whitney U | M meta & dia n=9  F meta & diap n=6 | meta U=25.50  dia U=22.50 | meta p=0.859 dia p=0.846 |
| Archivally identified M v. F juvenile+adolescent δ^13^C | Mann-Whitney U | M n=5  F n=11 | U=24.50 | p=0.732 |
| Archivally identified post-neonate to 6-year-old δ^15^N | Pearson correlation | meta n=45  dia n=46 | meta r=-0.200  dia r=-0.141 | meta 0.898  dia 0.349 |
| Archivally identified post-neonate to 6-year-old δ^13^C | Pearson correlation | meta n=45  dia n=46 | meta r=0.196  dia r=0.222 | meta 0.197  dia 0.139 |
| Archivally identified juveniles & adolescents δ^15^N | Pearson correlation | n=16 | r=-0.099 | 0.716 |
| Archivally identified juveniles & adolescents δ^13^C | Pearson correlation | n=16 | r=0.149 | 0.581 |
| Archivally identified adult F δ^15^N | Pearson correlation | n=66 | r=-0.013 | 0.917 |
| Archivally identified adult F δ^13^C | Pearson correlation | n=66 | r=-0.079 | 0.529 |
| Archivally identified adult M δ^15^N | Pearson correlation | n=53 | r=0.099 | 0.478 |
| Archivally identified adult M δ^13^C | Pearson correlation | n=53 | r=0.029 | 0.826 |
| All archivally identified adults δ^15^N | Pearson correlation | n=119 | r=0.450 | 0.625 |
| All archivally identified adults δ^13^C | Pearson correlation | n=119 | r=-0.059 | 0.522 |

Meta=metaphysis; Dia=diaphysis

M=male; F=female

YA=young adult (18-34 years); MA=middle adult (35-49 years); OA=old adult (50+ years)
